# Mesothelin-targeting T cell receptor fusion construct cell therapy in refractory solid tumors: phase 1/2 trial interim results

**DOI:** 10.1038/s41591-023-02452-y

**Published:** 2023-07-27

**Authors:** Raffit Hassan, Marcus Butler, Roisin E. O’Cearbhaill, David Y. Oh, Melissa Johnson, Kevin Zikaras, Munisha Smalley, Michael Ross, Janos L. Tanyi, Azam Ghafoor, Nirali N. Shah, Babak Saboury, Liang Cao, Alfonso Quintás-Cardama, David Hong

**Affiliations:** 1 Thoracic and GI Malignancies Branch, Center for Cancer Research, National Cancer Institute, National Institutes of Health, Bethesda, MD USA; 2 Princess Margaret Cancer Centre, Toronto, ON Canada; 3 Department of Medical Oncology, Memorial Sloan Kettering Cancer Center, Weill Cornell Medical College, New York, NY USA; 4 Division of Hematology/Oncology, Department of Medicine, University of California, San Francisco, San Francisco, CA USA; 5 Sarah Cannon Cancer Center, Nashville, TN USA; 6 TCR2 Therapeutics, Cambridge, MA USA; 7 Hospital of the University of Pennsylvania, Abramson Cancer Center, Philadelphia, PA USA; 8 Pediatric Oncology Branch, Center for Cancer Research, National Cancer Institute, National Institutes of Health, Bethesda, MD USA; 9 Department of Radiology and Imaging Sciences, Clinical Center, National Institutes of Health, Bethesda, MD USA; 10 Genetics Branch, Center for Cancer Research, National Cancer Institute, National Institutes of Health, Bethesda, MD USA; 11 Department of Investigational Cancer Therapeutics, The University of Texas MD Anderson Cancer Center, Houston, TX USA

**Keywords:** Mesothelioma, Immunotherapy, Ovarian cancer, Cancer immunotherapy, Phase I trials

## Abstract

The T cell receptor fusion construct (TRuC) gavocabtagene autoleucel (gavo-cel) consists of single-domain anti-mesothelin antibody that integrates into the endogenous T cell receptor (TCR) and engages the signaling capacity of the entire TCR upon mesothelin binding. Here we describe phase 1 results from an ongoing phase1/2 trial of gavo-cel in patients with treatment-refractory mesothelin-expressing solid tumors. The primary objectives were to evaluate safety and determine the recommended phase 2 dose (RP2D). Secondary objectives included efficacy. Thirty-two patients received gavo-cel at increasing doses either as a single agent (*n* = 3) or after lymphodepletion (LD, *n* = 29). Dose-limiting toxicities of grade 3 pneumonitis and grade 5 bronchioalveolar hemorrhage were noted. The RP2D was determined as 1 × 10^8^ cells per m^2^ after LD. Grade 3 or higher pneumonitis was seen in 16% of all patients and in none at the RP2D; grade 3 or higher cytokine release syndrome occurred in 25% of all patients and in 15% at the RP2D. In 30 evaluable patients, the overall response rate and disease control rate were 20% (13% confirmed) and 77%, respectively, and the 6-month overall survival rate was 70%. Gavo-cel warrants further study in patients with mesothelin-expressing cancers given its encouraging anti-tumor activity, but it may have a narrow therapeutic window. ClinicalTrials.gov identifier: NCT03907852.

## Abstract (web summary)

T cells containing an anti-mesothelin single-domain antibody fused to a component of the endogenous T cell receptor signaling complex exhibit notable toxicities but encouraging clinical responses in patients with treatment-refractory mesothelioma and ovarian cancer.

## Main

Mesothelin is a cell surface glycoprotein that is normally expressed on mesothelial cells lining the pleura, peritoneum and pericardium^1^. However, mesothelin overexpression occurs in approximately 30% of human cancers^2,3^, which makes it an attractive tumor antigen for targeted cancer therapy^4–8^, including T-cell-based treatments^9^.

Adoptive cell therapy using T cells engineered to express chimeric antigen receptors (CARs) has shown remarkable efficacy in several hematologic malignancies^10–15^. However, with few exceptions^16–18^, CAR-T cell therapies, including those targeting mesothelin, have been largely ineffectual in solid tumors^19–23^.

**Fig. 1:**
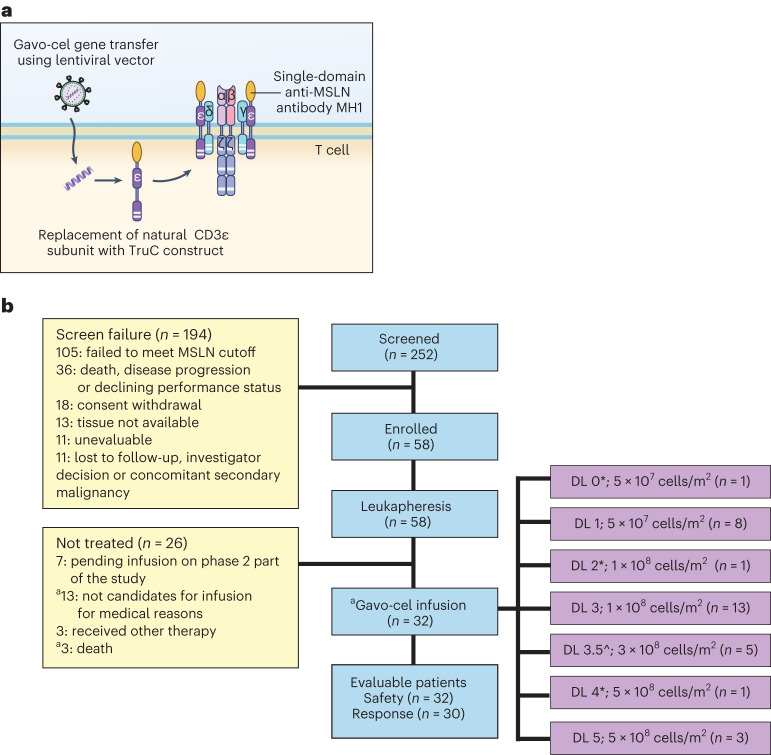
Gavo-cel construct and CONSORT diagram. **a**, Schematic diagram of gavo-cel. The patient’s autologous T cells are transduced with lentivirus carrying a plasmid encoding for an anti-mesothelin, llama-derived, single-domain antibody (MH1) fused to the CD3ε subunit using a linker sequence. Upon translation, the gavo-cel transgene integrates into the native TCR complex, exploiting the native TCR-driven T cell activation, effector function and regulation. **b**, CONSORT diagram showing patients enrolled and treated at different DLs. ^*^ Without lymphodepletion; ^a^ ITT patient population—patients who signed informed consent and underwent leukapheresis with the intent to receive gavo-cel as part of the phase 1 portion of the study; ^^^ Split dose of 1 × 10^8^ and 2 × 10^8^ cells on days 0 and 3. MSLN, mesothelin.

CAR-T cell constructs include only one (CD3ζ) of the six distinct T cell receptor (TCR) subunits tethered to a co-stimulatory domain (for example, CD28 or 4-1BB) and are expressed as standalone signaling receptors in transduced T cells, physically and functionally removed from native TCRs^24^. Gavocabtagene autoleucel (gavo-cel; TC-210), a T cell receptor fusion construct (TRuC), results from the fusion of the humanized, llama-derived, single-domain anti-mesothelin antibody MH1 to a 15-amino-acid flexible glycine/serine spacer (+G4S)3 and the human CD3ε subunit. The gavo-cel TRuC construct is then cloned into a lentiviral backbone, which, upon transduction into T cells, integrates into and reprograms native CD3 complexes to become activated upon recognition of tumor cell surface mesothelin in a human leukocyte antigen (HLA)-independent manner (Fig. 1a)^25^. Leveraging the signaling capacity of the entire TCR in this manner results in improved T cell trafficking, long-term functional persistence and enhanced anti-tumor activity of the TRuC compared to CAR T cells of the same specificity in a variety of murine models of human cancer^26^. Here we describe results of the recently completed phase 1 part of an ongoing phase1/2 clinical trial of gavo-cel in patients with mesothelin-expressing solid tumors (ClinicalTrials.gov: NCT03907852).

## Results

### Clinical trial design

This trial was an open-label, first-in-human, multicenter phase 1 study in patients with treatment-refractory, mesothelin-expressing cancers. The primary objective was to evaluate safety and determine the recommended phase 2 dose (RP2D) of gavo-cel. Secondary objectives included efficacy, by determining overall response rate (ORR), disease control rate (DCR), time to response (TTR) and T cell kinetics. Patients with pathologically confirmed malignant pleural or peritoneal mesothelioma (MPM), non-small cell lung cancer (NSCLC), ovarian cancer or cholangiocarcinoma were eligible if they had progressive disease despite standard of care therapy; if they had an Eastern Cooperative Oncology Group (ECOG) performance status of 0 or 1; and if their tumors expressed mesothelin in ≥50% of tumor cells with a 2+ and/or 3+ intensity, as determined by central immunohistochemistry (IHC) assessment.

**Extended Data Fig. 1:**
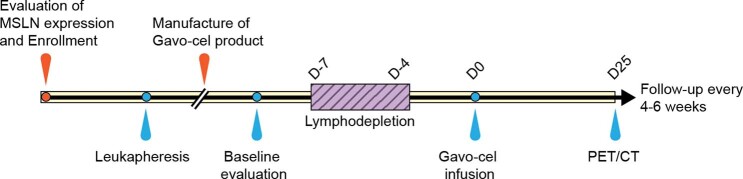
Schematic representation of the Gavo-cel study design. Tumor biopsies were evaluated for mesothelin (MSLN) expression at enrolment, followed by leukapheresis and gavo-cel manufacture. The enrolled patients underwent lymphodepletion with fludarabine at 30 mg/m^2^/day, from days -7 to -4 and cyclophosphamide at 600 mg/m^2^/day from days -6 to -4. On day 0, they were infused with different doses of gavo-cel. Positron emission tomography (PET) and computerized tomography (CT) scans were performed at baseline, and at 1, 2, 3, 6 and 9, and 12 months after gavo-cel infusion.

Gavo-cel was infused at different dose levels (DLs) either as a single agent or after lymphodepletion (LD) with fludarabine and cyclophosphamide (Extended Data Fig. 1). A detailed description of the study design and main eligibility criteria is included in the Methods section.

Between May 2019 and May 2022, 252 patients were screened at seven participating centers across the United States. The dates for the first and the last enrolled patients were 17 June 2019 and 28 February 2022, respectively. Of these, 58 eligible participants underwent large-volume leukapheresis, of whom 48 did so with the intent to receive a gavo-cel infusion in phase 1 (intent-to-treat (ITT) population). A gavo-cel product was manufactured for 56 of 58 apheresed patients (Fig. 1b). Peripheral blood mononuclear cells (PBMCs) were isolated, and frozen cells were shipped to a central facility for gavo-cel production by lentiviral transduction of the TRuC construct. Thirty-two patients received a single gavo-cel infusion at one of seven DLs (Fig. 1b). Participants enrolled to DL0, DL2 and DL4 did not receive LD. The remaining 29 patients received an LD regimen consisting of fludarabine 30 mg/m^2^ on days −7 through −4 and cyclophosphamide 600 mg/m^2^ on days −6 through −4. Patients enrolled to DL3.5 received 3 × 10^8^ transduced cells per m^2^ in two fractions of 1 × 10^8^ and 2 × 10^8^ transduced cells per m^2^ given on days 0 and 3, respectively. All patients infused with gavo-cel have been followed for safety, efficacy and gavo-cel T cell kinetics for at least 12 weeks. The median follow-up time was 17.3 months (range, 3.2–28.3 months) from the start of protocol-defined therapy. The cutoff date for data analysis was 9 September 2022.

### Patient characteristics at baseline

Thirty-two patients were infused with gavo-cel, including 23 with mesothelioma, eight with ovarian cancer and one with cholangiocarcinoma. Patient characteristics at baseline are listed in Table 1. The median age of patients who received gavo-cel was 63 years (range, 28–84 years) and included 12 males and 20 females. The median number of tumor cells expressing mesothelin with a 2+/3+ intensity was 70% (range, 50–100%) (Supplementary Fig. 1). The median number of prior therapies was five (range, 1–13), including immune checkpoint inhibitors (ICIs) in 21 patients (66%) and other mesothelin-directed therapies in six patients (19%). Among the 23 patients with malignant mesothelioma, 20 (87%) had progressive disease (PD) after at least one line of prior ICI therapy, including nine (39%) patients who had received two or more ICI agents. Bridging therapies were given to 24 patients (75%) while waiting for gavo-cel T cell engineering and product release. Thirteen patients were treated at the RP2D (six MPM, six ovarian cancers and one cholangiocarcinoma).

**Table 1:** Baseline characteristics of patients treated with gavo-cel

DL	DL0*	DL1	DL2*	DL3	DL3.5	DL4*	DL5	Overall
Gavo-cel dose (cells per m^2^)	5 × 10^7^	5 × 10^7^	1 × 10^8^	1 × 10^8^	3 × 10^8^	5 × 10^8^	5 × 10^8^	-
Number of patients	1	8	1	13	5	1	3	32
Age, median (range)	61	70 (36–84)	46	59 (28–70)	63 (43–69)	67	52 (37–66)	63 (28–84)
Diagnosis	1 MPM	7 MPM, 1 ovarian	1 MPM	6 MPM, 6 ovarian, 1 CHO	4 MPM, 1 ovarian	1 MPM	3 MPM	23 MPM, 8 ovarian, 1 CHO
Mesothelin 2+/3+	90	72 (55–100)	90	70 (50–95)	75 (50–92)	60	65 (65–73)	70 (50–100)
Median number of prior therapies (range)	8	5	9	5	7	7	4	5 (1–13)
Prior ICI, *n* (%)	1 (100)	6 (75)	1 (100)	6 (46)	4 (80)	1 (100)	2 (66)	21 (66)
Prior anti-mesothelin therapy, *n* (%)	1 (100)	1 (13)	1 (100)	1 (8)	2 (40)	0	1 (33)	6 (19)

^*^ No LD.

CHO, cholangiocarcinoma.

### Safety of gavo-cel

The primary objective of this study was to evaluate the safety of gavo-cel through 28 d after infusion. All treatment-related adverse events (AEs) reported in at least 10% of patients are listed in Extended Data Table 1. As shown in Table 2, the most frequent AEs of grade 3 or higher were cytopenias related to lymphodepleting chemotherapy, including 91% of patients with neutropenia, 97% with lymphopenia and 22% with thrombocytopenia, which were reversible in all cases. On-target/off-tumor toxicities observed were pleuritis (grade 3 (*n* = 2) at DL 3 and DL3.5), pericarditis (grade 3 (*n* = 1) at DL3.5) and peritonitis (grade 3 (*n* = 1) at DL3).

**Table 2:** Treatment-emergent AEs of special interest (grade 3 and higher)

AEs	DL0 (%)	DL1 (%)	DL2 (%)	DL3 (%)	DL3.5 (%)	DL4 (%)	DL5 (%)	Overall (%)
Gavo-cel dose (cells per m^2^)	5 × 10^7^/m^2^*	5 × 10^7^/m^2^	1 × 10^8^/m^2^*	1 × 10^8^/m^2^	3 × 10^8^/m^2^	5 × 10^8^/m^2^*	5 × 10^8^/m^2^	
Number of patients	1	8	1	13	5	1	3	32
Hematologic toxicity								
Lymphopenia	1 (100)	8 (100)	0	13 (100)	5 (100)	1 (100)	3 (100)	31 (97)
Neutropenia	0	8 (100)	0	13 (100)	5 (100)	0	3 (100)	29 (91)
Thrombocytopenia	0	2 (25)	0	2 (15)	1 (20)	0	2 (67)	7 (22)
On-target/on-tumor								
CRS	0	2 (25)	0	2 (15)	1 (20)	0	3 (100)	8 (25)
On-target/off-tumor								
Pericarditis/pericardial effusion	0	0	0	0	1 (20)	0	0	1 (3)
Pleuritis/pleural effusion	0	0	0	1 (8)	1 (20)	0	0	2 (6)
Peritonitis/ascites	0	0	0	1 (8)	0	0	0	1 (3)
Other								
Pneumonitis	0	1 (13)	0	0	3^§+^ (60)	0	1 (33)	5 (16)
Sepsis	0	1 (13)^§++^	0	0	0	0	0	1 (3)
Hemorrhage	0	0	0	0	0	0	1 (33)^§+++^	1 (3)
Respiratory failure	0	0	0	0	0	0	1 (33)^§++++^	

All emergent AEs described in the table were deemed at least possibly related to protocol-defined therapy.

^*^ No LD.

^§^ Grade 5 AEs: ^+^One patient developed respiratory failure possibly related to gavo-cel; ^++^patient developed fungal sepsis unrelated to gavo-cel; ^+++^patient had bronchioalveolar hemorrhage after CRS-related disseminated intravascular coagulation; ^++++^respiratory failure in patient with concomitant CMV pulmonary infection and pneumothorax.

Cytokine release syndrome (CRS) was reported in 25 (78%) patients, being grade 1 or 2 in 17 (53%), grade 3 in six (19%) and grade 4 in two (6%). No clear relationship was observed between tumor type and CRS incidence or severity. The median onset of CRS was 3 d (range, 0–9 d) and lasted for a median of 6 d (range, 1–55 d). CRS was managed with tocilizumab in 17 patients, including 11 patients who also required corticosteroids and two patients who required siltuximab. Eight patients with CRS did not require any anti-cytokine therapy. No grade 5 CRS was observed. Per the American Society for Transplantation and Cellular Therapy (ASTCT) criteria^27^, one patient developed a brief and self-limited case of immune effector cell-associated neurotoxicity syndrome (ICANS).

Two dose-limiting toxicities (DLTs) were reported. The first one was a grade 3 pneumonitis event experienced by a patient with mesothelioma treated at DL1, in the context of grade 3 CRS. Both complications resolved with tocilizumab and corticosteroid therapy, but several weeks thereafter, the patient developed grade 5 fungal sepsis, which was deemed unrelated to gavo-cel therapy. Because of the pneumonitis event, DL1 was initially expanded from three patients to six patients, upon which dose escalation proceeded to DL2. The second DLT was a grade 5 bronchioalveolar hemorrhage event reported in a patient with mesothelioma treated at DL5 who had previously developed CRS-related disseminated intravascular coagulation and was on prophylactic dose of low-molecular-weight heparin therapy at the time of the hemorrhagic event. Two additional deaths were reported in the study. A patient treated at DL5 developed grade 5 respiratory failure in the setting of grade 3 CRS and concomitant pneumothorax and cytomegalovirus (CMV) pulmonary infection. In addition, a patient treated at DL3.5 developed pneumonitis and grade 5 respiratory failure, possibly related to gavo-cel therapy.

A trend toward dose dependency was observed regarding incidence, severity and onset of CRS, with all three patients treated at DL5 developing grade 3 or higher CRS (reported within the first 24 h after infusion). This led the Safety Review Team to stop dose escalation at this level and to recommended de-escalation to 3 × 10^8^/m^2^ (DL3.5), to be administered in a fractionated manner: 1 × 10^8^ cells per m^2^ on day 0 and 2 × 10^8^ cells per m^2^ on day 3 after LD. However, three of five patients at DL3.5 developed grade 3 or higher pneumonitis, which led the Safety Review Team to declare DL3 (1 × 10^8^ cells per m^2^ after LD) as the RP2D. Although, numerically speaking, the dose differential between DL3 (RP2D) and DL3.5 appears moderate, the toxicity profiles observed at each of these DLs were markedly different in terms of the frequency and severity of both pneumonitis and CRS. No cases of pneumonitis were reported at the RP2D, and only two patients developed severe CRS, which was rapidly reversible in both cases. This is in contrast with DL3.5, where three of five patients developed pneumonitis, and at DL5, where all patients developed severe CRS, which developed more rapidly, lasted longer and was less responsive to anti-cytokine therapy. These data delineate a therapeutic window for gavo-cel and highlight the importance of monitoring patients for signs of CRS or pneumonitis.

Of 13 safety-evaluable patients at the RP2D, eight had grade 3/4 gavo-cel-related AEs, for a rate of 61% (95% confidence interval (CI), 44–77%), including reversible grade 3 or higher CRS in three (15%) patients after a median of 3 d (range, 2–4 d) and no pneumonitis events.

### Anti-tumor efficacy of gavo-cel

**Fig. 2:**
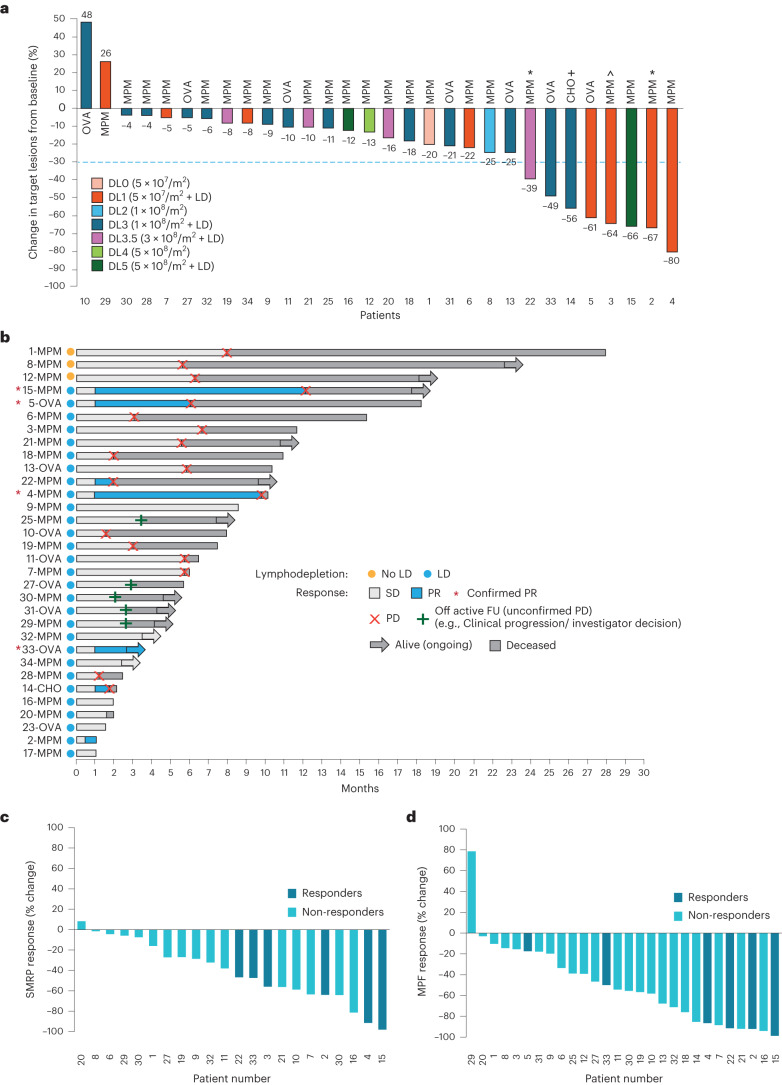
Efficacy of gavo-cel treatment. **a**, Waterfall plot showing maximum change in target lesion size from baseline in evaluable patients (*n* = 30) as assessed by BICR. Bars are colored according to DLs. ^*^ Patients 2 and 22 had an unconfirmed PR; ^+^ patient 14 had an unconfirmed PR by local investigator assessment; ^^^ patient 3 showed more than 30% regression of target lesions on a 6-month CT scan but also a new lesion that prevented the patient from achieving a PR. CHO, cholangiocarcinoma; OVA, ovarian cancer. **b**, Swimmer plot showing best responses of each patient over time, assessed according to RECIST version 1.1. ^*^ confirmed PR. **c**,**d**, Changes in circulating surrogate markers of tumor response. Waterfall plots showing best response (% change from baseline) and SMRPs as measured in plasma using the MESOMARK assay (**c**) and MPF as measured in plasma using an ELISA assay (**d**). Responders (partial or complete response) and non-responders (SD and PD) are indicated by color-coded bars. FU, follow-up. Source data

**Extended Data Fig. 2:**
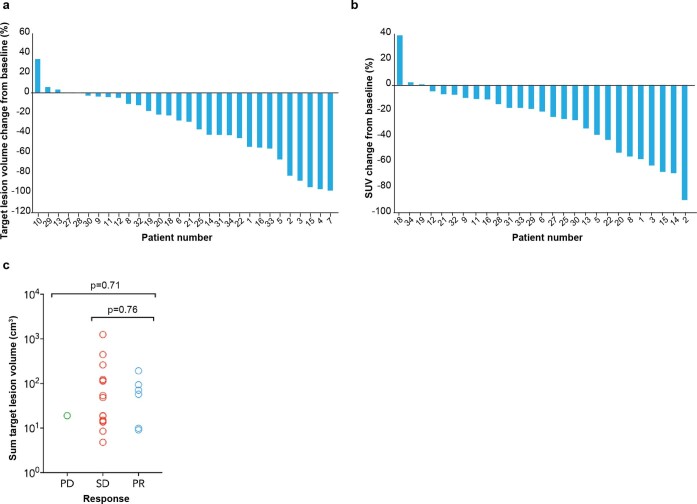
Tumor Response to gavo-cel treatment. **a–b, Volumetric and Metabolic Response to gavo-cel treatment. a, Volumetric analysis**. Maximum reduction of sum of volumes of target lesions post gavo-cel infusion. **b, Metabolic response analysis**. Maximum reduction of standardized uptake value (SUV) of the target lesion with the highest SUV assessed at the baseline visit (that is prior to gavo-cel infusion). **c, Baseline target lesion volume and response to gavo-cel treatment**. Baseline target lesion volume does not correlate with radiological response assessment. The *p* values were calculated using the Kruskal-Wallis test for n = 29 patients who were treated with gavo-cel.

Of the 32 patients who received gavo-cel, 30 were evaluable for tumor response assessment. Two patients who experienced grade 5 AEs before their post-infusion computed tomography (CT) scan were not evaluable for response. After gavo-cel infusion, all but two patients (93%) experienced a decrease in the sum of target lesion diameters, ranging in magnitude from 4% to 80% (Fig. 2a). Target lesion regression was even deeper when assessed by volumetric analysis and was coupled with metabolic responses by positron emission tomography (PET) (Extended Data Fig. 2a,b). Patients 1, 8 and 12, who received gavo-cel without prior LD, also experienced tumor regression, resulting in prolonged treatment-free intervals. However, greater tumor regression was observed among patients receiving gavo-cel after LD, with eight patients (five with MPM, two with ovarian cancer and one with cholangiocarcinoma) experiencing more than a 30% reduction in tumor size. Tumor regression of target lesions appeared to be independent of the pre-infusion tumor burden (Extended Data Fig. 2c).

**Extended Data Fig. 3:**
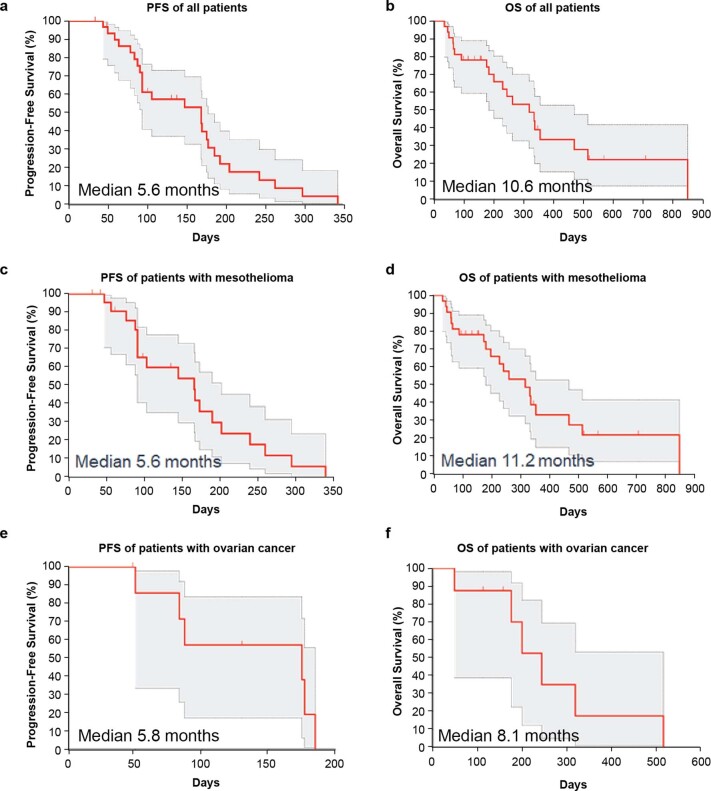
Survival after gavo-cel treatment. **a-f**. PFS and OS curves were estimated by the Kaplan-Meier method. n = 32 patients infused with gavo-cel, including 30 who completed at least 12 weeks of follow-up visits. **a**, Progression Free Survival in all patients (5.6 months, 95% CI, 3.1, 5.8, n = 32). **b**, Overall survival in all patients. (10.6 months, 95% CI, 6.6, 15.6) **c**, Progression Free Survival in patients with mesothelioma (5.6 months, 95% CI, 3.1, 5.8, n = 23). **d**, Overall survival in patients with mesothelioma (11.2 months, 95% CI, 6.0, 15.6). **e**, Progression Free Survival in patients with ovarian cancer (5.8 months, 95% CI, 1.6, 6.1, n = 8). **f**, Overall survival in patients with ovarian cancer (8.1 months, 95% CI, 1.6, 17.1).

The DCR, as determined by blinded independent central review (BICR), was 77%. The ORR according to Response Evaluation Criteria in Solid Tumors (RECIST) version 1.1 was 20% by BICR (12.5% in the ITT population), which included six patients (four with MPM and two with ovarian cancer) (Fig. 2b). Of these six patients, four had a confirmed partial response (PR) (13%; two MPM and two ovarian cancer). By local investigator assessment, the ORR was 23% (15% in the ITT population), which included one additional patient with cholangiocarcinoma who was also considered to have achieved a PR. One additional patient with mesothelioma had a gradual response, with target lesions regressing less than 30% at 3 months. The 6-month CT scan showed target lesion tumor regression of more than 30%; however, a new lesion was also identified, and, therefore, the patient was not considered to have a PR. The ORR by BICR and by local investigator assessment was 22% and 26%, respectively, among patients who received gavo-cel after LD. The median time to response (TTR) was 1.15 months (95% CI, 0.76–1.53), whereas the median duration of response (DoR) was 4.6 months (95% CI, 1.77–7.42). The median progression-free survival (PFS) and overall survival (OS) for all patients was 5.6 months (95% CI, 3.1–5.8) and 10.6 months (95% CI, 6.6–15.6), respectively, and the 6-month OS was 70.2% (Extended Data Fig. 3a,b). For patients with MPM, the ORR among those receiving gavo-cel after LD was 21%. This clinical activity resulted in a median PFS of 5.6 months (95% CI, 3.1–5.8) and median OS of 11.2 months (95% CI, 6.0–15.6) (Extended Data Fig. 3c,d). For patients with ovarian cancer, the ORR among those who received gavo-cel after LD was 29%, with a median PFS of 5.8 months (95% CI, 1.6–6.1) and a median OS of 8.1 months (95% CI, 1.6–17.1) (Extended Data Fig. 3e,f).

**Extended Data Fig. 4:**
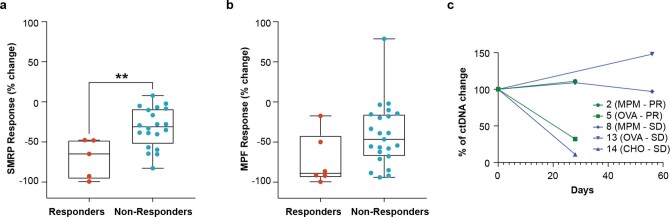
Changes in circulating surrogate tumor markers correlate with radiographic tumor response. SMRP was measured in plasma using the Mesomark™ assay and MPF was measured in plasma using an ECL assay and ctDNA was measured using a plasma based NGS assay. Comparative analysis of **a**, best SMRP response (n = 25 independent patient samples, **p = 0.074 by two-tailed Mann Whitney t test. and **b**, best MPF response in responders vs. non-responders. (n = 29 independent patient samples). For both a, and b, patients classified as non-evaluable by RECIST response were excluded from the analysis. For both box-plots; center line, box limits and whiskers represent the median, interquartile range and minima and maxima, respectively. **c**, Longitudinal changes in ctDNA levels in selected patients. Source data

Serum levels of soluble mesothelin-related peptides (SMRPs) and megakaryocytic potentiating factor (MPF) were monitored throughout the study. Both markers have been proposed as surrogate markers of clinical activity in clinical trials of anti-mesothelin agents^28,29^. SMRP response was evaluable in 22 of 31 patients who showed baseline SMRP levels above the normal range (>1.5 nM). After treatment, 21 of 22 of these patients showed decreases from baseline SMRP levels, with significantly deeper SMRP responses in patients with deep radiological responses to gavo-cel (Fig. 2c and Extended Data Fig. 4a). Likewise, MPF levels (evaluated in 29/31 patients) also showed post-treatment decreases from baseline with a trend toward greater decreases in patients who responded to gavo-cel (Fig. 2d and Extended Data Fig. 4b).

In addition, a post hoc circulating tumor DNA (ctDNA) analysis was assessed in 11 patients using the Guardant Health OMNIpanel; samples from five of 11 patients harbored mutations that allowed for ctDNA analysis (Extended Data Fig. 4c). Clearance of ctDNA was observed in two of the five patients.

### Durable tumor response in a patient with mesothelioma

**Fig. 3:**
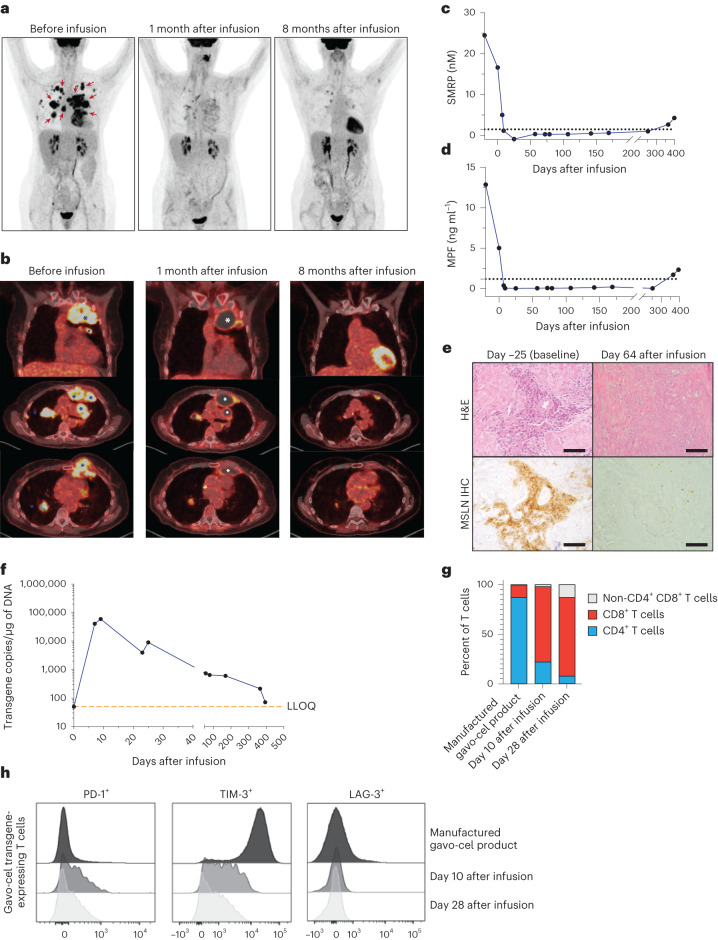
Tumor regression in a patient (patient 15) with MPM after gavo-cel treatment. **a**, Frontal view of ^18^F-FDG PET maximum intensity projection obtained before infusion, approximately 1 month after infusion and 8 months after infusion. Areas of tumor involvement are indicated by red arrows. **b**, Representative PET–CT images obtained at the same timepoints as in **a**. Top part of **b** is coronal image at level of ascending aorta; middle part is axial section at level just below carina; and lower part is axial section at level of base of heart. Tumor areas are indicated by blue asterisk, and fluid-filled regions after tumor regression are marked with white asterisk. **c**,**d**, Circulating surrogate tumor response biomarker. **c**, Decrease in SMRP after gavo-cel infusion. **d**, Decrease in MPF level after infusion. **e**, Mesothelin-specific cell killing in post-infusion tumor biopsy. Tumor biopsies obtained at enrollment and 64 d after gavo-cel infusion were stained with hematoxylin and eosin (H&E) (top). Mesothelin expression was evaluated by IHC (bottom). H&E staining indicated the presence of epithelioid malignant mesothelioma, with most tumor cells showing 3+ mesothelin staining intensity at baseline. The tumor biopsy obtained 8 weeks after gavo-cel infusion showed complete tumor necrosis with loss of mesothelin expression in dead cells. Experiment was performed once on patient samples collected at different timepoints. Inset scale bars, 100 µM. **f**, Persistence of gavo-cel in peripheral blood after infusion by qPCR. Peak expansion was observed at day −10, followed by a contraction phase that plateaued approximately 60 d after infusion, with gavo-cel T cells remaining detectable at the latest measurement: 1 year after infusion. **g**, Phenotypic analysis of gavo-cel product and post-infusion kinetics. Proportion of CD4^+^ and CD8^+^ T cell subsets in manufactured gavo-cel product and in the gavo-cel transgene-expressing T cells obtained from peripheral blood of the patient after infusion. **h**, Exhaustion markers in gavo-cel manufactured product and in the gavo-cel transgene-expressing T cells obtained from the patient’s peripheral blood after the infusion. Expression of PD-1, TIM-3 and LAG-1 in manufactured gavo-cel product, on gavo-cel transgene-expressing T cells on days 10 and 28 after infusion. MSLN, mesothelin. Source data

**Extended Data Fig. 5:**
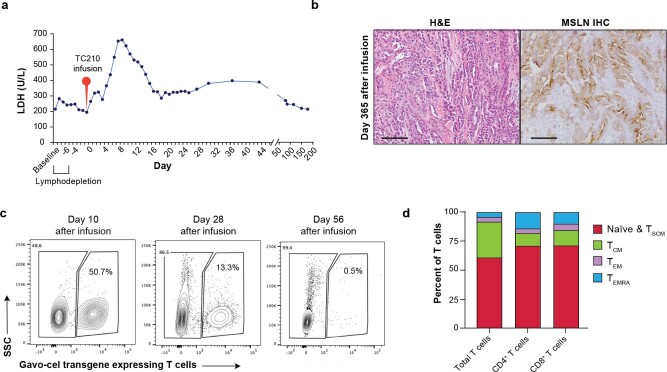
Response in a patient with malignant pleural mesothelioma. **a, Systemic inflammation following gavo-cel treatment. a**, Increase in serum lactate dehydrogenase (LDH) after infusion of gavo-cel that peaked at day 9. It gradually decreased to reach normal range by day 142 (normal range: 125–220 U/mL). **b, Mesothelin expression in tumor biopsy after progression**. Tumor biopsy obtained after progression on day +365 shows mesothelin expression. Experiment performed once on patient samples. Inset scale bars represents 100 µM. **c, Persistence of gavo-cel in peripheral blood post-infusion by flow cytometry**. Flow cytometry analysis of PBMCs isolated from peripheral blood and stained with anti-VHH antibody showed 50.7% cells being TRuC+ on day 10. On day 28 this was reduced to 13.3%, whereas on day 56, 0.5% cells were detected as TRuC+. **d, Phenotypic analysis of gavo-cel product**. Flow-cytometric analysis of the different T cell subsets in the manufactured gavo-cel product that was infused into the patient. Phenotyping revealed a predominance of stem cell memory (T_SCM_) and naïve T (T_N_) cells, with the majority of the remaining T cells having a central memory T cell (T_CM_) phenotype. The proportion of T_SCM_ and T_N_ was similar in the CD4+ and CD8 + T cell subsets. T_EM_, T effector memory cell; T_EMRA_, effector memory T cells re-expressing CD45RA. Source data

The kinetics of tumor response with continued tumor regression after a single gavo-cel infusion is illustrated by patient 15, a 67-year-old female with metastatic epithelioid MPM with treatment-refractory disease after progression on four prior lines of therapy. She was infused 5 × 10^8^/m^2^ gavo-cel T cells (total dose, 8.8 × 10^8^ transduced T cells) after LD. The patient manifested early signs of tumor cell killing as evidenced by increase in serum lactate dehydrogenase (LDH) levels starting on day 5 after gavo-cel infusion that peaked on day 9 and gradually decreased thereafter (Extended Data Fig. 5a). A CT scan on day +25 showed a significant decrease in the size of the tumor masses, which were now mostly fluid filled, coupled with a remarkable reduction of ^18^F-fluorodeoxyglucose (FDG) uptake that subsequently almost completely resolved. Subsequent CT scans showed continuous shrinkage or complete resolution of the tumor masses, which was observed for 12 months, at which point she developed disease progression (Fig. 3a,b and Supplementary Fig. 2a,b). The patient’s elevated baseline SMRP and MPF levels decreased below the level of detection by day +9 and remained undetectable until progression, day +365 (Fig. 3c,d). A biopsy of the chest wall mass (Fig. 3b, bottom panel) on day 64 after infusion showed complete tumor necrosis with no evidence of mesothelin expression (Fig. 3e). A biopsy obtained 25 d before gavo-cel infusion showed the presence of malignant mesothelioma, epithelioid type, with most tumor cells showing strong positive membranous mesothelin staining (Fig. 3e). A biopsy taken on day +365 at time of tumor progression showed high mesothelin expression, suggesting that antigen loss was not a reason for the progression of her tumor (Extended Data Fig. 5b).

As shown in Fig. 3f and Extended Data Fig. 5c, the gavo-cel transgene remained detectable in peripheral blood at the last measurement: 1 year after infusion. The manufactured gavo-cel product consisted predominantly of T stem cell memory (T_SCM_) and naive T (T_N_) cells, with most of the remaining T cells having a central memory T cell (T_CM_) phenotype. The proportion of T_SCM_ and T_N_ was similar for the CD4^+^ and CD8^+^ T cell subsets (Extended Data Fig. 5d). Phenotypically, the TRuC-transduced T cells in the manufactured gavo-cel product were predominantly CD4^+^ (Fig. 3g). However, samples obtained from the patient 2 weeks and 4 weeks after gavo-cel infusion showed that most of the circulating T cells were predominantly CD8^+^ (Fig. 3g). The manufactured gavo-cel product expressed low levels of PD-1 and very high levels of TIM-3, but PD-1 expression on gavo-cel T cells increased more than fourfold by day 10 after infusion and decreased to pre-infusion levels by day 28. On the other hand, TIM-3 expression on gavo-cel decreased by day 10 after infusion and remained low when analyzed on day 28. LAG-3 expression on gavo-cel product was negligible both before and after infusion (Fig. 3h). Although factors leading to gavo-cel persistence need more study, responses to gavo-cel could possibly be due to the fact that the infused gavo-cel product had very low expression of T cell exhaustion markers and a high proportion of T_N_ and T_SCM_ cells—a T cell population associated with proliferation and long-term T cell persistence^30^.

**Extended Data Fig. 6:**
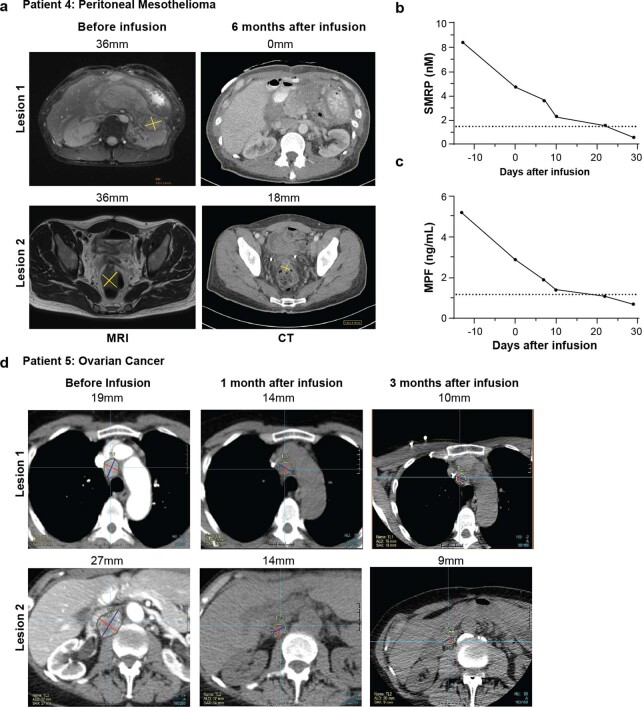
Tumor response in patients with MPM and ovarian cancer. **Tumor regression in a 36-year-old male with epithelioid malignant peritoneal mesothelioma treated at dose level 1 after checkpoint inhibitor failure**. **a**, Representative MRI images at baseline and CT scan images 6 months post gavo-cel infusion showing complete tumor regression of lesion abutting the left kidney (lesion 1) and reduction in size of the tumor in the peritoneal reflection of the rectum (lesion 2). **b–c, Circulating surrogate biomarkers of tumor response. b**, Decrease in serum SMRP and **c**, MPF levels following gavo-cel infusion. **Tumor regression in a 70-year-old female with platinum refractory high grade serous ovarian cancer treated at dose level 1. d**, Representative CT scan images show regression in target lesion size of mediastinal lymph node (lesion 1) and abdominal retro-caval lymph node (lesion 2) at 1- and 3- months after gavo-cel infusion and maintained up to 6 months post treatment. The measurements denote the size of the target lesions Source Data.

Other examples of durable radiologic response seen in patients with mesothelioma and ovarian cancer are shown in Extended Data Fig. 6.

### Gavo-cel-induced cytokine release and correlation with CRS

**Fig. 4:**
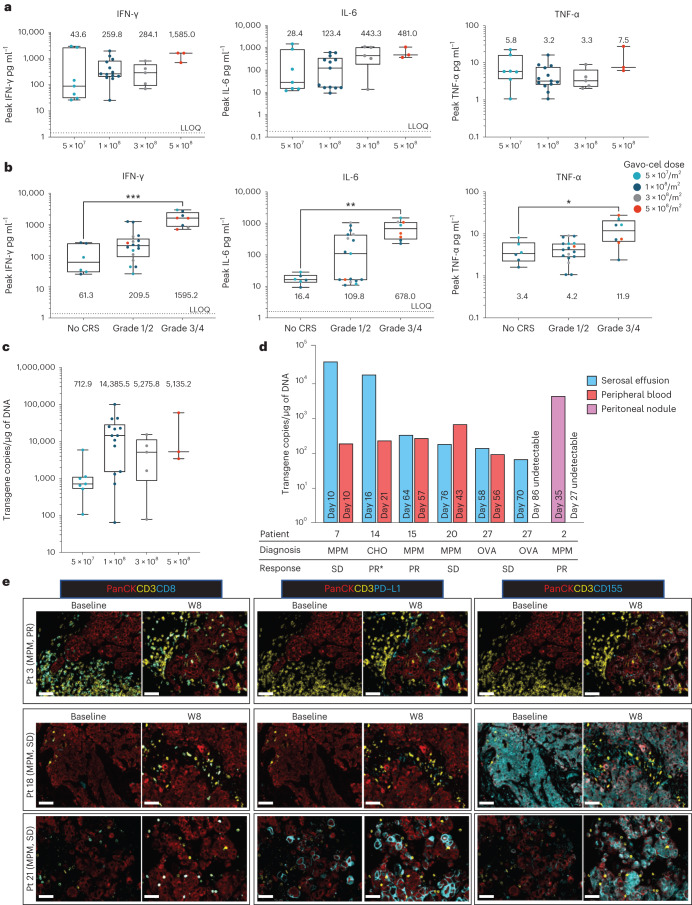
Cytokine response, gavo-cel expansion and persistence. **a**,**b**, Peak cytokine response by DL and correlation of peak cytokine levels with CRS. Plasma cytokine levels were measured longitudinally in the peripheral blood using a validated multiplexed immunoassay (MSD). Horizontal lines and boxes show the medians and interquartile ranges. **a**, Peak levels of IFN-γ, IL-6 and TNF-α after gavo-cel infusion in patients who received LD (for the 5 × 10^7^/m^2^ cohort, *n* = 7 independent patient samples; for the 1 × 10^8^/m^2^ cohort, *n* = 13 independent patient samples; for the 3 × 10^8^/m^2^ cohort, *n* = 5 independent patient samples; for the 5 × 10^8^/m^2^ cohort, *n* = 3 independent patient samples). **b**, Correlative analysis of peak IFN-γ, IL-6 and TNF-α levels with grade of CRS (for the no-CRS cohort, *n* = 6 independent patient samples; for the grade 1/2 cohort, *n* = 17 independent patient samples; for the grade 3/4 cohort, *n* = 8 independent patient samples; statistical significance was determined by one-way Kruskal−Wallis ANOVA: IFN-γ ***P = 0.0003; IL-6 **P = 0.02; TNF-α *P = 0.002). **c**,**d**, Expansion and persistence of gavo-cel. Gavo-cel expansion was monitored by qRT–PCR **c**, Peak expansion (C_max_) levels of gavo-cel T cells in peripheral blood by DL shows maximum expansion in DL 1 × 10^8^/m^2^ (for the 5 × 10^7^/m^2^ cohort, *n* = 7; for the 1 × 10^8^/m^2^ cohort, *n* = 14; for the 3 × 10^8^/m^2^ cohort, *n* = 5; for the 5 × 10^8^/m^2^ cohort, *n* = 3). For box plots: center line, box limits and whiskers represent the median, interquartile range and minima and maxima, respectively. **d**, Expansion of gavo-cel T cells in malignant serosal effusions, peripheral blood and peritoneal nodule. Experiment was performed once on each independent patient sample. CHO, cholangiocarcinoma; OVA, ovarian cancer; PR^*^, partial response by investigator assessment. **e**, Characterization of TME before and after gavo-cel infusion by multiplex immunofluorescence. Multiplex immunofluorescent staining was performed for cytokeratin (PanCK, tumor marker), CD3 (pan-T cell marker), CD8, PD-L1 and CD155 in MPM tumor biopsies taken at baseline and at week 8 after gavo-cel infusion from patient 3, who achieved a PR by best target lesion response, and patients 18 and 21, both having a best response of SD. Experiment was performed once on each independent patient sample. Scale bars, 50 µM. Pt, patient; W, week. Source data

After infusion, serum levels of IFN-γ, IL-6, TNF-α, IL-8 and IL-10 generally increased compared to baseline levels (Supplementary Fig. 3). However, IL-12, IL-2, IL-4, IL-13 and IL-1β were not clearly detected after gavo-cel infusion (Supplementary Fig. 4). Peak plasma cytokine levels of IFN-γ, IL-6 and TNF-α showed a trend toward dose dependency (Fig. 4a), and the peak levels of these cytokines also correlated with increased risk of developing a higher-grade CRS (Fig. 4b).

### Manufacture and characteristics of gavo-cel product

A single apheresis was sufficient to manufacture a gavo-cel product meeting the protocol-specified doses for all patients infused on the study. The median time from leukapheresis to gavo-cel production start was 11 d (range, 3–260 d). The median transduction efficiency was 49% (range, 33–80%), and the median vector copy number in the manufactured product was 2.47 per cell (range, 1.19–6.67).

**Extended Data Fig. 7:**
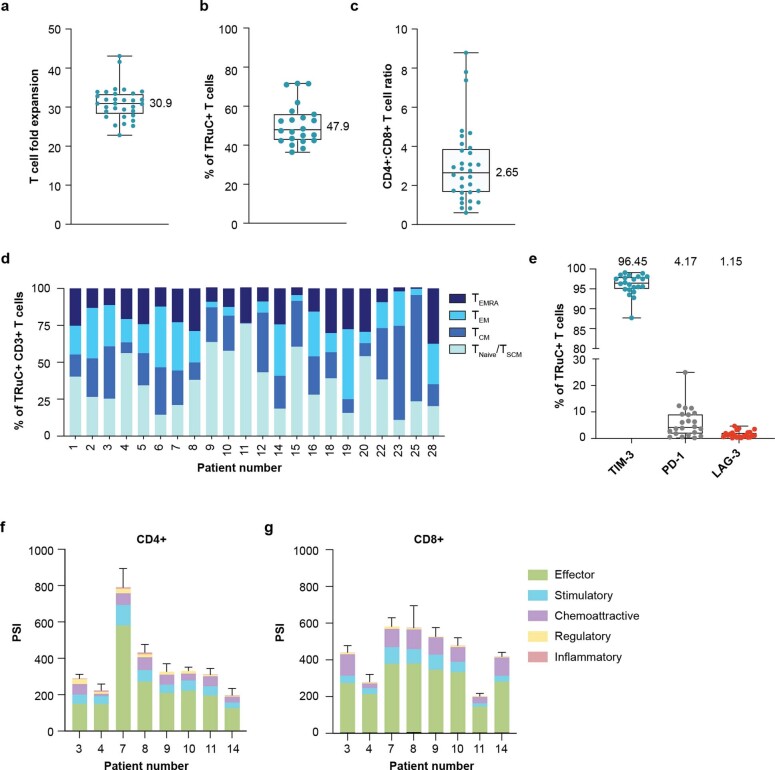
Characterization of gavo-cel T cell products (TCPs). Flow cytometry methods were used to characterize TCPs. **a**, Median fold expansion of the TCPs during the manufacturing process was 30.9 (n = 32 independent patient samples). **b**, Percent of TRuC+ T cells was 47.9% (n = 22 independent patient samples). **c**, The ratio of CD4+ to CD8 + T cells was 2.65 (n = 32 independent patient samples). For box-plots, center line, box limits and whiskers represent the median, interquartile range and minima and maxima, respectively. **d**, Memory subset composition using the markers CD45RA and CCR7 showed variable composition of stem cell memory (T_SCM_) and naïve T cells, central memory T cell (T_CM_), T effector memory cell (T_EM_) and effector memory T cells re-expressing CD45RA (T_EMRA_ subsets). **e**, Expression of the exhaustion markers TIM-3, PD-1, and LAG-3 were determined by flow-cytometry. TIM-3 expression was high, PD-1 was variable, and LAG-3 was low. (n = 22 independent patient samples) For box plots; center lines, box limits and whiskers represent the median, interquartile range and minima and maxima, respectively. **f–g, Gavo-cel T cell products TCPs show robust polyfunctionality**. Multiplexed assessment of cytokine production at the single cell level was performed by Isoplexis assay in isolated **f**, CD4+ (n = 8 independent patient samples) and **g**, CD8 + T cells within gavo-cel TCPs. (n = 8 independent patient samples) Polyfunctionality of T cells defined as a cell co-secreting 2 or more cytokines were analyzed by the IsoSpeak software across the seven functional groups. The polyfunctional strength Index (PSI) of T cells was defined as the percentage of polyfunctional cells, multiplied by the sum of the mean fluorescence intensity (MFI) of the proteins secreted by those cells: PSI = (% polyfunctional cells in sample) x ∑(MFI of all 32 secreted proteins of the polyfunctional cells).TCP predominantly secreted effector, stimulatory, and chemo attractive proteins Source. Source data

The median T cell fold expansion during the manufacturing process was 30.9 (range 22.8–43.1) (Extended Data Fig. 7a), and the median percentage of cells positive for TRuC expression was 47.9% (range 36.4–71.6%) (Extended Data Fig. 7b). The CD4^+^-to-CD8^+^ T cell ratio was highly variable across gavo-cel products, with a median of 2.65 (range, 0.86–27.14) (Extended Data Fig. 7c). The memory subset composition was also highly variable (Extended Data Fig. 7d). Exhaustion/activation marker analysis of the manufactured gavo-cel product revealed ubiquitous expression of TIM-3, variable expression of PD-1 and low to negligible expression of LAG-3 (Extended Data Fig. 7e). The high expression of TIM-3 in the gavo-cel product is likely due to the use of IL-7 and IL-15 during the manufacturing process, as was previously described^31^, but it decreased upon infusion into patients.

The polyfunctionality of selected gavo-cel products (*n* = 8) was assessed using the single-cell IsoPlexis assay to measure the secretion of 32 cytokines at the single-cell level. After 24 h of stimulation with plate-bound mesothelin, all evaluated gavo-cel products showed robust polyfunctionality, with polyfunctional strength index (PSI) scores (an overall cellular potency metric) greater than 200 in both CD4^+^ and CD8^+^ subsets, which secreted predominantly effector, stimulatory and chemoattractive proteins (Extended Data Fig. 7f,g).

### Gavo-cel expansion, persistence and tumor infiltration

**Extended Data Fig. 8:**
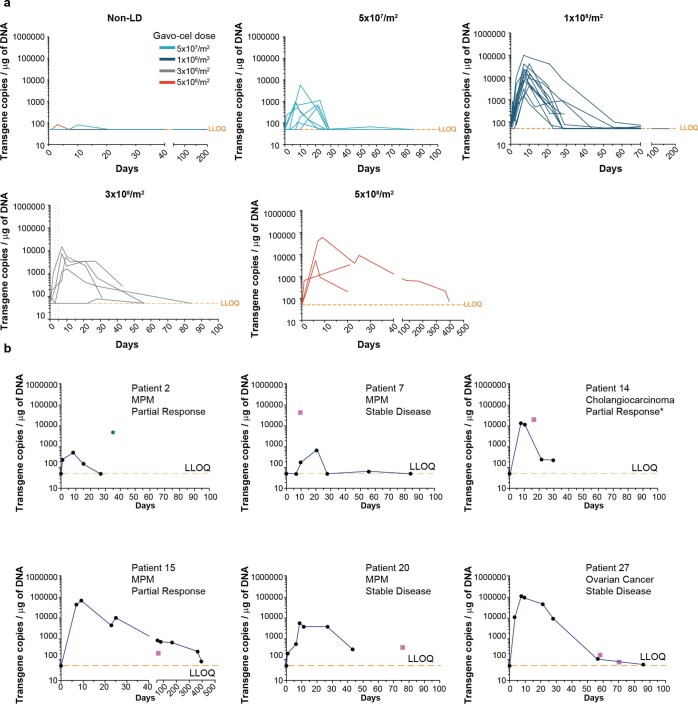
Expansion and persistence of gavo-cel in peripheral blood. **a**, Kinetics of gavo-cel T cell expansion and persistence in peripheral blood by qPCR-based detection of specific TRuC-encoding proviral sequences in genomic DNA. Patients are grouped by dose level. **b**, Gavo-cel expansion and persistence over time in peripheral blood and at specific time points in cancerous tissues. Pink squares denote gavo-cel transgene level in serosal effusion; green circle denotes gavo-cel transgene level in peritoneal mesothelioma nodule. *Partial response by local investigator assessment. Source Data

The post-infusion expansion and persistence kinetics of gavo-cel are displayed in Fig. 4c and Extended Data Fig. 8a. In the patients (*n* = 3) who did not receive LD, expansion in the peripheral blood was minimal (below the lower limit of quantitation (LLOQ) of 50 copies per microgram (µg) of genomic DNA (gDNA) in one of three patients) and short-lived. After LD, expansion was greatly increased with median C_max_ of 712.9 copies per µg of gDNA (range, 50–5,900.8) at the 5 × 10^7^/m^2^ dose, 14,385.45 (range, 50–100,781.9) at the 1 × 10^8^/m^2^ dose, 5,135.2 (range, 50–15,320.0) at the 3 × 10^8^/m^2^ dose and 5,275.8 (range, 50–59,542.3) at the 5 × 10^8^/m^2^ dose (Fig. 4c). At the RP2D, the median peripheral blood persistence was 84 d (range, 0–170 d). C_max_ was not associated with achieving a RECIST response to treatment (Supplementary Data Fig. 5). In addition, some patients—for example, patients 4 and 5 as shown in Fig. 2a—either achieved an objective response or had a continued deepening response months after the infused gavo-cel T cells had become undetectable in peripheral blood by both flow cytometry and qPCR.

Trafficking of gavo-cel into tumor tissue or malignant effusions was evaluated in five patients. Tumor lesions collected at autopsy from patient 2 at 34 d after infusion showed clearly detectable levels of TRuC-T cell infiltration in two of five analyzed lesions: a peritoneal nodule (4,928 copies per μg of gDNA) and a heart nodule (4,473 copies per μg of gDNA). Notably, the gavo-cel transgene had become undetectable in peripheral blood by day 27, and no transgene was detected in normal lung adjacent to tumor lesions. Serosal effusion samples collected after infusion from four patients showed clearly detectable levels of TRuC-T cells. The peritoneal tumor nodule from patient 2, and effusions from patients 7 and 14, showed a much higher TRuC-T cell detection signal when compared to blood samples collected at the nearest proximal timepoint and at the time of peak expansion (Fig. 4d, Extended Data Fig. 8b and Supplementary Table 1). These data indicate that gavo-cel expansion occurs preferentially within cancerous tissues with high mesothelin density, where engineered T cells can be detected long after they have become undetectable in peripheral blood.

### Changes in the tumor microenvironment after treatment

To gain insight into the potential impact of gavo-cel therapy on the tumor microenvironment (TME), we analyzed pre-treatment and post-treatment biopsies from three patients with MPM—one patient who experienced a PR (patient 3, 64% reduction in target lesion size) and two patients with a best response of stable disease (SD) (patients 18 and 21). At baseline, the TME of the responding patient was characterized by an abundance of T cells that were clearly excluded from tumor cell nests. At 8 weeks after treatment, T cells were observed to robustly infiltrate into the tumor of this responding patient. In the two non-responders, T cells were less abundant at baseline, and levels of infiltration did not notably change after treatment (Fig. 4e, left panel, and Supplementary Fig. 6a). Expression of the immunoinhibitory ligand PD-L1 was increased after treatment in all three patients, with the highest level of increase observed in non-responder patient 21 whose tumor showed the highest basal level of PD-L1 expression (Fig. 4e, central panel, and Supplementary Fig. 6b). Interestingly, expression of a second immunoinhibitory ligand, CD155, which functionally interacts with TIGIT, was observed to be high at both baseline and post-treatment in non-responder patient 18 and was markedly upregulated after treatment in non-responder patient 21. In contrast, levels of CD155 were undetectable at baseline in the responder, with only a minor increase in staining intensity observed after treatment (Fig. 4e, right panel, and Supplementary Fig. 6c). These preliminary observations suggest that gavo-cel tumor infiltration may result in the remodeling of the TME, with upregulation of immunoinhibitory ligands that may represent a potential mechanism of resistance.

**Extended Data Fig. 9:**
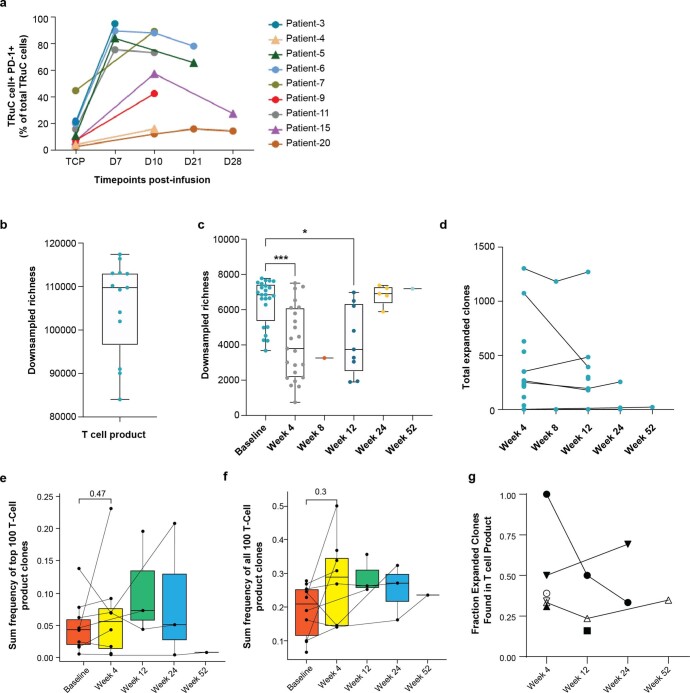
PD1 expression and T cell clonotypic expansion in blood post-gavo-cel infusion. **a, PD1 expression**. Percent of PD1 positive gavo-cel T cells in the manufactured T cell product (TCP) and at different time-points post infusion. Circles denote subjects with stable disease whereas triangles denote subjects that achieved a partial response. These data show that irrespective of response, PD-1 expression on gavo-cel T cells increases post-infusion. **b–g, Clonotypic analysis was performed by TCRB CDR3 sequencing. b**, Downsampled richness as a measure of clonal diversity in analyzed TCPs. (n = 13 independent patient samples) **c**, Analysis of downsampled richness in the blood at baseline and longitudinally for all available subjects post-infusion. (n = 30 subjects, with varying longitudinally sample availability, baseline vs. week 4 ***p = 0.001, baseline vs. week 12 * p = 0.0473) **d**, Longitudinal analysis of total expanded clones for all available patients. **e–f**, Top 100 T cell product (TCP) clones and the sum frequency of clones found in peripheral blood over time (up to 52 weeks) are shown for all analyzed subjects. (n = 8 subjects, with varying longitudinally sample availability) **g**, Proportion of expanded clones found in the TCP. (n = 12 independent patients, with varying longitudinally sample availability). For c, e, f Statistical significance was determined by Kruskal-Wallis ANOVA and a Dunn’s multiple comparison to determine the p-value. For box-plots; center line, box limits and whiskers represent the median, interquartile range and minima and maxima, respectively. Source Data

Furthermore, we also evaluated the kinetics of gavo-cel PD1 expression after infusion. For the nine patients in whom this was evaluated in a longitudinal manner, there was an increase in PD-1 expression on gavo-cel T cells after infusion, compared to the pre-infusion gavo-cel product. This increase in PD-1 expression was seen irrespective of tumor response (Extended Data Fig. 9a). These findings further support the use of gavo-cel in combination with nivolumab in the ongoing phase 2 trial.

### T cell clonotypic expansion

Clonotypic analysis revealed a high level of clonal diversity in gavo-cel T cell products (TCPs) (Extended Data Fig. 9b). Clonotypic T cell expansion assessed longitudinally in the blood of selected patients showed a significant decrease in clonality at 4 weeks after gavo-cel infusion, followed by a gradual trend toward increased clonality suggesting the expansion of dominant clones (Extended Data Fig. 9c). Total expanded clones were traceable longitudinally (Extended Data Fig. 9d). The frequency of the TCP clones remained around 15–30%, which suggests that variations in T cell clone richness was likely not related to the limited persistence of gavo-cel in peripheral blood (Extended Data Fig. 9e,f). Furthermore, when analyzed longitudinally, approximately 40% of the expanding clones from the baseline sample involved clones found in the TCP, although, after week 4, the number of analyzed samples is limited (Extended Data Fig. 9g).

### Immunogenicity assessment

Anti-drug antibody (ADA) levels were assayed to detect the formation of antibodies against the anti-mesothelin MH1 sdAb binder contained in the gavo-cel TRuC. There were no instances of pre-existing ADA, and only one of 28 evaluated patients displayed ADA positivity after infusion (Supplementary Table 2).

## Discussion

The results of our study demonstrate the safety and feasibility of administering gavo-cel, a first-in-class TRuC-targeting mesothelin, in patients with mesothelin-expressing solid tumors. To our knowledge, this is the first report of an engineered T cell therapy inducing clinical responses by leveraging the activation of the TCR complex in an HLA-unrestricted manner.

This study is also noteworthy because, to our knowledge, gavo-cel is the first anti-mesothelin adoptive T cell therapy to show consistent objective radiologic responses and one of few studies to show tumor regression in patients with solid tumors^16–18^. Several anti-mesothelin CAR-T cell approaches have been tested in clinical trials for patients with solid tumors, including T cells engineered to transiently express an mRNA-encoded mesothelin-specific CAR^19^ as well as T cells stably transduced with an anti-mesothelin lentiviral construct^21^. Both strategies proved safe but had limited clinical activity. A recent trial administered anti-mesothelin CAR-T cells locoregionally through a pleural catheter or directly into the tumor^23^. Although no objective radiologic responses were seen in the 23 patients with mesothelioma who received these CAR-T cells after cyclophosphamide preconditioning, two patients had a PR after they were treated with pembrolizumab off protocol.

As expected, the most frequently reported AEs in this study were cytopenias related to the administration of LD. The most frequent non-hematological AE was CRS, which was reported in 78% of patients, being grade 3 or higher in 25% of patients. However, gavo-cel at the RP2D (1 × 10^8^/m^2^ after LD) was associated with significant activity and a manageable safety profile, thus lending itself to a combination approach with ICIs, which is currently ongoing in the phase 2 portion of the study.

A single gavo-cel infusion resulted in radiologic tumor regression in 93% of patients, and the ORR among patients with mesothelioma or ovarian cancer receiving gavo-cel after LD was 21% and 29%, respectively, as assessed by BIRC. This is significant given that the median number of prior therapies was five, including at least one line of standard ICI therapy in 87% of patients with MPM. Notably, objective responses were observed only in patients who received gavo-cel after LD, which led to T cell expansion and persistence in a dose-dependent fashion, and induction of cytokine secretion, thus highlighting the importance of the conditioning regimen.

In addition to inducing radiological responses, gavo-cel appeared to prolong survival in a heavily pre-treated patient population. The PFS and OS in MPM were 5.6 months and 11.2 months, respectively. These results are encouraging, as patients with MPM who fail ICI therapy are typically treated with chemotherapy regimens. In the second-line setting, such approaches render an ORR lower than 10%, a PFS of ~3 months and an OS of ~10 months^32–34^. Nevertheless, these results should be interpreted with caution given the implicit selection biases built into the design of cellular therapy phase 1 clinical trials and the absence of a comparator arm.

A potential limitation of gavo-cel therapy is the fact that responses, regardless of depth, were, in some cases, of limited duration. Antigen escape and T cell exhaustion have been invoked to explain disease relapse in patients with hematological malignancies receiving adoptive T cell therapies^35^. Although mesothelin expression was still present in biopsies obtained 8 weeks after infusion, this may be too early a timepoint to assess antigen loss. On the other hand, an increase in PD-1 expression on gavo-cel T cells was observed during the first 28 d after infusion and may represent a harbinger of gavo-cel exhaustion, thus justifying the combination of gavo-cel with anti-PD-1 agents, similar to the experience with anti-mesothelin CAR-T cell therapy^23^. Such an approach could result in increased gavo-cel persistence in peripheral blood, which, at the RP2D, was approximately 30–40 d. This could further increase gavo-cel persistence in the TME, where we showed high levels of gavo-cel T cells long after they had become undetectable in peripheral blood. Treatment-emergent T-cell-mediated anti-TRuC responses were not assessed, but they might have also limited gavo-cel persistence.

Our data strongly support the development of TRuC-based cell therapies in patients with solid tumors as well as mesothelin as a target. Given the promising preliminary results reported here, a phase 2 study is underway to evaluate the efficacy of gavo-cel in mesothelin-expressing cancers, which will allow for repeat intravenous dosing and the combination with ICI therapy as strategies to promote greater gavo-cel exposure and to prevent exhaustion of the engineered T cells.

## Methods

### Study design

This is a phase 1/2, single-arm, open-label, multicenter, first-in-human clinical trial (NCT03907852) designed to evaluate the safety and efficacy of gavo-cel therapy in patients with mesothelin-expressing recurrent or metastatic malignant mesothelioma, ovarian cancer, lung adenocarcinoma or cholangiocarcinoma. The primary objective of the phase 1 study was to evaluate safety and determine the RP2D of gavo-cel. The secondary objective was to access efficacy by determining ORR, DCR, TTR, DoR, PFS and OS. Patients with a response include those who achieved a partial or complete response, whereas non-responders are those with SD or PD. Exploratory objectives were to correlate response with gavo-cel expansion, persistence, phenotype and functionality. Gavo-cel tumor infiltration (before and after evaluation of the TME), measurement of immune cell markers and correlation with clinical response to treatment were also studied.

For the phase 1 dose-escalation part of the study, patients received a single dose of intravenous gavo-cel infusion with or without LD. The protocol-specified DLs were 1 × 10^7^, 5 × 10^7^, 1 × 10^8^, 3 × 10^8^, 5 × 10^8^ and 1 × 10^9^ transduced cells per m^2^. A maximum of 48 patients could be treated during the dose-escalation phase. DLs without LD required the enrollment of only one patient to proceed to dose escalation in the absence of significant toxicity, whereas DLs employing LD required the enrollment of three patients and proceeded following a standard 3 + 3 dose-escalation schema. Patients were enrolled regardless of sex and/or gender, which was determined by patient self-reporting. An analysis based on sex/gender was not performed.

Informed consent was obtained from all participants in the clinical trial, as stated in the clinical trial protocol. Participants were not compensated for their participation in the clinical trial. Neither sex nor gender was considered in the study design. Patients were enrolled on a ‘first come, first served’ basis. Sex/gender was determined based on self-report. The trial was approved by the institutional review board of the participating centers, and all patients provided written informed consent. The seven participating sites are: Thoracic and GI Malignancies Branch, Center for Cancer Research, National Cancer Institute, National Institutes of Health, Bethesda, MD, USA; Princess Margaret Cancer Centre, Toronto, ON, Canada;Department of Medical Oncology, Memorial Sloan Kettering Cancer Center, Weill Cornell Medical College, New York, NY, USA; Division of Hematology/Oncology, Department of Medicine, University of California, San Francisco, San Francisco, CA, USA; Sarah Cannon Cancer Center, Nashville, TN, USA; Hospital of the University of Pennsylvania, Abramson Cancer Center, Philadelphia, PA, USA; and Department of Investigational Cancer Therapeutics, The University of Texas MD Anderson Cancer Center, Houston, TX, USA. This trial was sponsored by TCR^2^ Therapeutics.

### Patient eligibility

Eligibility for the study required that patients met all of the following criteria:

1. Voluntarily agreed to participate by giving written informed consent in accordance with International Conference on Harmonization (ICH) Good Clinical Practice (GCP) guidelines and applicable local regulations.
2. Agreement to abide by all protocol-required procedures, including study-related assessments, and management by the treating institution for the duration of the study and long-term follow-up.
3. Age ≥18 years at the time the informed consent is signed.
4. Pathologically confirmed diagnosis of MPM, serous ovarian adenocarcinoma (patients with serous fallopian tube or primary peritoneal cancers were also eligible), cholangiocarcinoma or NSCLC.
5. Tumor was pathologically reviewed at a sponsor-designated central laboratory with confirmed positive mesothelin expression on ≥50% of tumor cells with 2+ and/or 3+ staining intensity by IHC. A fresh biopsy for confirmation of mesothelin expression was required at baseline (if not done at pre-screening) before gavo-cel administration.
6. Patient had advanced (that is, metastatic or unresectable) cancer.
7. Patient had at least one lesion that met evaluable and measurable criteria defined by RECIST version 1.1.
8. Before gavo-cel infusion, patients must have received at least one systemic standard of care therapy for metastatic or unresectable disease and have failed FDA-approved agents for their disease (for example, PARP inhibitors for *BRCA1/2-*mutated ovarian cancer or osimertinib for patients with *EGFR T790M* mutation). Patients with newly diagnosed cholangiocarcinoma could receive gavo-cel infusion if they elected not to pursue frontline standard of care therapy.
9. ECOG performance status 0 or 1.
10. Negative rapid influenza diagnostic test and/or a respiratory viral panel (as per institutional guidelines) within 14 d before the planned gavo-cel infusion. Respiratory viral panel should be performed according to institutional guidelines and include Coronavirus Disease 2019 (COVID-19; severe acute respiratory syndrome coronavirus 2 (SARS-CoV-2)). If the patient was symptomatic or tested positive, the gavo-cel infusion was delayed until the patient was asymptomatic and deemed fit for infusion by the treating physician.
11. Left ventricular ejection fraction ≥45% as measured by resting echocardiogram, with no clinically significant pericardial effusion.
12. Females of childbearing potential (FCBPs) must have a negative urine or serum pregnancy test (FCBP is defined as premenopausal and not surgically sterilized). FCBPs must agree to use effective birth control or to abstain from heterosexual activity throughout the study, starting on the day of first dose of lymphodepleting chemotherapy through 12 months after gavo-cel infusion or for 4 months after there is no evidence of persistence of gene-modified cells in the blood, whichever is longer. Effective contraceptive methods include intra-uterine device, oral or injectable hormonal contraception or two adequate barrier methods (for example, diaphragm with spermicide, cervical cap with spermicide or female condom with spermicide). Spermicides alone are not an adequate method of contraception. Male patients must be surgically sterile or agree to use a double-barrier contraception method or abstain from heterosexual activity with an FCBP starting at the first dose of protocol-defined treatment and for 4 months thereafter or longer (if indicated in the country-specific monograph/label for cyclophosphamide).
13. Patient must have adequate organ function according to the following laboratory values:

**Table:** 

Hematological	
Absolute neutrophil count	≥1 × 10^9^/L (without growth factor support)
Absolute lymphocyte count	≥0.3 × 10^9^/L
Platelets	≥100 × 10^9^/L
Hemoglobin	≥90 g L^−1^ (without transfusion support within 7 d before protocol-defined therapy)
Coagulation	
Prothrombin time	≤1.5× upper limit of normal (ULN)
Partial thromboplastin time	≤1.5× ULN
Renal	
Creatinine clearance (Cockcroft–Gault formula)	≥40 ml min^−1^
Hepatic	
Serum total bilirubin	≤2× ULN (unless patient has documented Gilbert’s syndrome or unless secondary to bile duct obstruction by tumor)
Alanine aminotransferase	≤2.5× ULN or ≤5× ULN if documented liver metastasis
Aspartate aminotransferase	≤2.5× ULN or ≤5× ULN if documented liver metastasis

Patients meeting any of the following criteria were not eligible for participation in the study:

1. Inability to follow the study procedures.
2. Known or suspected non-compliance or drug or alcohol abuse.
3. Participation in another study with investigational drug within the 28 d or 5 half-lives of the drug, whichever is shorter, preceding and during the present study.
4. Patient is pregnant (or intends to become pregnant during the course of the study) or breastfeeding.
5. Patient has received the following treatment before initiating protocol-defined therapy with either LD or gavo-cel:
  i. Cytotoxic chemotherapy within 3 weeks of gavo-cel infusion.
  ii. Corticosteroids: therapeutic doses of steroids must be stopped at least 2 weeks before gavo-cel infusion. Inhaled corticosteroids are not exclusionary.
  iii. Immunosuppression: any other immunosuppressive drugs (for example, methotrexate and mycophenolate) must be stopped ≥4 weeks before the first protocol-defined treatment.
  iv. Use of an anti-cancer vaccine within 2 months in the absence of tumor response or 6 months if responding.
  v. Any previous gene therapy using an integrating vector.
  vi. Tyrosine kinase inhibitors within 72 h.
  vii. Any previous allogeneic hematopoietic stem cell transplant.
  viii. Investigational treatment or clinical trial within 4 weeks or 5 half-lives of investigational product, whichever is shorter.
  ix. Radiotherapy to the target lesions within 3 months before lymphodepleting chemotherapy unless palliative radiotherapy to non-targeted lesions.
  x. Current anti-coagulative therapy (excluding deep vein thrombosis prophylaxis).
  xi. Immunotherapy (monoclonal antibody therapy and checkpoint inhibitors) within 4 weeks.
6. Toxicity from previous anti-cancer therapy that had not recovered to ≤grade 1 (except for non-clinically significant toxicities—for example, alopecia and vitiligo). Grade 2 toxicities that are deemed stable or irreversible (for example, peripheral neuropathy) are non-exclusionary.
7. History of allergic reactions attributed to compounds of similar chemical or biologic composition to fludarabine, cyclophosphamide or other agents used in the study.
8. History of autoimmune or immune-mediated disease, such as multiple sclerosis, lupus, rheumatoid arthritis, inflammatory bowel disease or small vessel vasculitis.
9. Major surgery (other than diagnostic surgery) within 4 weeks before the first protocol-defined therapy and minor surgery, including diagnostic surgery, within 2 weeks (14 d), excluding central intravenous port placements and needle aspirate/core biopsies. Radiofrequency ablation or transcatheter arterial chemoembolization within 6 weeks before enrollment.
10. Leptomeningeal disease, carcinomatous meningitis or symptomatic central nervous system (CNS) metastases: patients are eligible if they have recovered from the acute effects of radiation therapy or surgery before study entry and (1) have no evidence of brain metastases after treatment or (2) are asymptomatic, have discontinued corticosteroid or anti-seizure therapy for metastases for at least 4 weeks and have radiographically stable CNS metastases (no growth, edema or shift for at least 3 months before study entry).
11. Any other prior or concurrent malignancy, with the exception of treated basal cell or squamous cell carcinoma, in situ carcinoma of the cervix or breast, stage 0 or 1 melanoma completely resected more than 12 months before enrollment, successfully treated organ-confined prostate cancer and other malignancies completely resected and in remission for more than 5 years.
12. Electrocardiogram showing a clinically significant abnormality at screening.
13. Uncontrolled intercurrent illness, including active infection, clinically significant cardiac disease (for example, congestive heart failure New York Heart Association class 3 or class 4, significant arrhythmia and acute coronary syndrome), interstitial lung disease, liver cirrhosis or primary sclerosing cholangitis.
14. Active infection with HIV, hepatitis B virus, hepatitis C virus or human T-lymphotropic virus.

### Gavo-cel manufacturing, LD and bridging therapy

PBMCs were collected via leukapheresis using standardized procedures and cryopreserved following a controlled-rate freezing process. Manufacturing of a gavo-cel product was started a median of 11 d after leukapheresis (one product was started 260 d after leukapheresis collection due to the need to remanufacture after the initial product bag was found to be damaged). The manufacture of the gavo-cel product was carried out using the functionally closed CliniMACS Prodigy automated cell processing system at the Miltenyi Biotec manufacturing facility in San Jose, California. There, the apheresis blood product was attached to the CliniMACS Prodigy system through a sterile weld. After separation by Ficoll-Paque density gradient centrifugation, CD4^+^ and CD8^+^ T cells were isolated by magnetic bead separation and activated with TransAct CD3/CD28 (Miltenyi Biotec). Then, T cells were transduced with a lentiviral vector encoding for the gavo-cel transgene, consisting of an anti-mesothelin, llama-derived, single-domain antibody (MH1) fused to the CD3ε subunit of TCR using a linker sequence^25^. T cells were then expanded in the presence of TexMACS media (Miltenyi Biotec), IL-7 and IL-15. The gavo-cel product was harvested and formulated after the cell quantity reached the required dose. Each lot underwent in-process and release testing. Upon release, patients were infused with a single dose of gavo-cel on day 0, within 4 d of completion of lymphodepleting chemotherapy. Re-infusion was not permitted during the phase 1 part of the study. Pre-conditioning consisted of fludarabine 30 mg/m^2^ on days −7, −6, −5 and –4 and cyclophosphamide 600 mg/m^2^ on days –6, –5 and –4. Patients treated at DLs without LD proceeded directly to gavo-cel infusion on day 0. Patients actively progressing or clinically symptomatic were allowed to receive bridging therapy during the period of gavo-cel manufacturing. The choice of bridging therapy was individualized at the investigator’s discretion based on the patient’s previous cancer therapy and clinical status.

### AEs and assessment of tumor response

AEs were assessed and graded according to the National Cancer Institute (NCI) Common Terminology Criteria for Adverse Events (CTCAE) version 5.0 on a five-point scale (grade 1–5). CRS and neurotoxicity were evaluated using the ASTCT Consensus Grading for Cytokine Release Syndrome and Neurologic Toxicity Associated with Immune Effector Cells^27^. Per protocol, a CT scan/MRI was obtained from each enrolled patient at the time of eligibility determination and then again between day −21 and day −8 from gavo-cel infusion to define their disease status at baseline. After gavo-cel infusion, patients were assessed for radiologic response by CT scan/MRI at 4 weeks, 8 weeks, 12 weeks, 24 weeks and every 3 months thereafter until disease progression, study completion or patient withdrawal. Efficacy assessments were conducted according to RECIST version 1.1 (ref. ^36^) by the study investigators as well as by BICR involving at least two independent expert radiologists.

### Mesothelin IHC

Mesothelin expression was centrally assessed in all patients by Ventana Medical Systems using a validated IHC assay performed on 3–5-mm-thick sections of formalin-fixed paraffin-embedded (FFPE) tumor tissue. Automated immunostaining was performed using the VENTANA MSLN (SP74) Dx Assay, which is a rabbit monoclonal primary antibody that binds to mesothelin in paraffin-embedded tissue sections. The specific anti-mesothelin staining was visualized using OptiView DAB IHC Detection Kit (760-700 / 0639500001). A second slide was stained with rabbit monoclonal negative control Ig (790-4795 / 06683380001). Mesothelin protein expression was determined using both the percentage of stained viable tumor cells and the staining intensity^37^. Study eligibility required biopsy tissue to express membranous mesothelin in at least 50% of the viable tumor cells at a staining intensity of 2+ or 3+.

### Gavo-cel detection by flow cytometry in peripheral blood

PBMCs were isolated from whole blood by density gradient centrifugation and cryopreserved. Thawed samples were stained with LIVE/DEAD Aqua (Thermo Fisher Scientific) and fluorochrome-conjugated antibodies specific for CD3-BUV395 (BD Biosciences, 564001, clone SK7, 1:50 dilution), CD4-BUV496 (BD Biosciences, 612936, clone SK3, 1:25 dilution), CD8-APC-Cy7 (BD Biosciences, 348793, clone SK1, 1:100 dilution), CD366/TIM3-PE (BD Biosciences, 565570, clone 7D3, 1:400 dilution), CD27-BV605 (BioLegend, 302829, clone O323, 1:12.5 dilution), CD45RA-BV711 (BioLegend, 304137, clone HI100, 1:400 dilution), CD45RO-Alexa-700 (BioLegend, 304217, clone UCHL1, 1:25 dilution), CCR7-PE-Cy7 (BioLegend, 353225, clone G043H7, 1:25 dilution), CD95-BV785 (BioLegend, 305645, clone DX2, 1:100 dilution), CD279/PD-1-BV421 (BioLegend, 329919, clone EH12.2H7, 1:25 dilution), CD223/LAG-3 BV650 (BioLegend, 369315, clone 11C3C65, 1:50 dilution) and an anti-VHH-AF488 for TRuC detection (GenScript, A01862, clone 96A3F5, 1:400 dilution). Flow cytometry was performed on an LSR flow cytometer (BD Biosciences). BD FACSDiva software version 9.0 was used to collect data, and the data were analyzed using FlowJo software version 10.8.1 (BD Biosciences). Viable (PBMC) cells were counted before acquisition by means of the automated Guava counting process using the Viacount Reagent (GuavaEasyCyte HT and GuavaSoft version 3.3). The gating strategy used for identifying TRuC T cells in post-infusion peripheral blood is shown in Supplementary Fig. 7. Cells were gated for singlets, lymphocytes, viable cells and CD3^+^VHH^+^ T cells (Supplementary Fig. 7a). CD3^+^VHH^+^ T cells were further defined into memory phenotypes (CM, central memory; EM, effector memory; TEMRA, effector memory cells re-expressing CD45RA) (Supplementary Fig. 7b). Additional CD3^+^VHH^+^ T cells were analyzed for expression of PD-1, LAG-3 and TIM-3 (Supplementary Fig. 7c).

### Gavo-cel detection by qPCR in peripheral blood

PBMCs were isolated from whole blood by density gradient centrifugation and cryopreserved as dry pellets. gDNA was extracted from PBMCs using the Qiagen DNA Blood Mini Kit. A duplex real-time PCR assay was performed using TaqMan probes and primers to amplify the oPRE sequence in the TRuC transgene in tandem with endogenous albumin. oPRE copy number (copies per µg of gDNA) was calculated against standard curves consisting of known concentrations of linear oPRE and albumin double-stranded DNA (dsDNA) fragments. All qPCR reactions were performed using the Applied Biosystems ViiA7 Real Time PCR System (Life Technologies). dsDNA used for standard and control material was synthesized using GeneArt Gene Synthesis (Thermo Fisher Scientific), and the sequences are shown below. The sequence used for albumin was derived from within exon 7 of human albumin, National Center for Biotechnology Information reference sequence NM_000477.6. oPRE dsDNA: 389-bp linear fragment

AATCAACCTCTGGATTACAAAATTTGTGAAAGATTGACTGATATTCTTAACTATGTTGCTCCTTTTACGCTGTGTGGATATGCTGCTTTATAGCCTCTGTATCTAGCTATTGCTTCCCGTACGGCTTTCGTTTTCTCCTCCTTGTATAAATCCTGGTTGCTGTCTCTTTTAGAGGAGTTGTGGCCCGTTGTCCGTCAACGTGGCGTGGTGTGCTCTGTGTTTGCTGACGCAACCCCCACTGGCTGGGGCATTGCCACCACCTGTCAACTCCTTTCTGGGACTTTCGCTTTCCCCCTCCCGATCGCCACGGCAGAACTCATCGCCGCCTGCCTTGCCCGCTGCTGGACAGGGGCTAGGTTGCTGGGCACTGATAATTCCGTGGTGTTGTC

Albumin dsDNA: 350-bp linear fragment

TTATTTCTGTATGTCCATTTTGAATTTTCTTATGAGAAATAGTATTTGCCTAGTGTTTTCATATAAAATATCGCATGATAATACCATTTTGATTGGCGATTTTCTTTTTAGGGCAGTAGCTCGCCTGAGCCAGAGATTTCCCAAAGCTGAGTTTGCAGAAGTTTCCAAGTTAGTGACAGATCTTACCAAAGTCCACACGGAATGCTGCCATGGAGATCTGCTTGAATGTGCTGATGACAGGGTAAAGAGTCGTCGATATGCTTTTTGGTAGCTTGCATGCTCAAGTTGGTAGAATGGATGCGTTTGGTATCATTGGTGATAGCTGACAGTGGGTTGAGATTGTCTTCTGT

### SMRP assay

Serum SMRPs were evaluated using the MESOMARK ELISA kit (Fujirebio Diagnostics), following the manufacturer’s instructions. Venous blood was drawn at defined time intervals as per the protocol and processed for serum. Serum samples were stored at –80 °C until the assay was performed. Patients who had baseline SMP <1.5 mM were removed from the analysis because this is the cutoff for healthy subjects (MESOMARK, https://www.aruplab.com/mesomark#overview).

### MPF assay

Serum MPF was evaluated using an electrochemiluminescence (ECL) immunoassay developed by the Molecular Targets Core, Center for Cancer Research (CCR), NCI, using ECL reagents from Meso Scale Discovery (MSD). The MPF capture antibody, sulfo-tag MPF detection antibody and MPF calibrator were developed at CCR, NCI^29,38^. Venous blood was drawn at defined time intervals as per the protocol and processed for serum. Serum samples were stored at –80 °C until the assay was performed.

### Plasma cytokine measurement

Baseline and post-infusion venous blood samples were taken from patients and processed for plasma. Plasma samples were stored at –80 °C until assays were performed. Plasma (25 µl) was analyzed for the following cytokines in singlicate: IFN-γ, IL-10, IL-12p70, IL-13, IL-1b, IL-2, IL-4, IL-6, IL-8 and TNF-α by multiplexed ELISA using the V-PLEX Plus Proinflammatory Panel 1 Human Kit (MSD) and conducted according to the manufacturer’s guidelines under Good Clinical Laboratory Practice (GCLP).

### IsoPlexis polyfunctionality assay

Patient-derived TRuC drug product was thawed and allowed to recover in complete TexMACS GMP Medium in a 37 °C, 5% CO_2_ incubator for 1 h. TRuC cells were enriched using biotinylated anti-camelid VHH antibody labeling (GenScript, A01995) anti-biotin MultiSort Kit (Miltenyi Biotec, 130-091-256) and anti-CD8 microbeads (Miltenyi Biotec, 130-045-201). Enriched TRuC CD4^+^ and CD8^+^ cells were incubated with plate-bound recombinant human mesothelin (10 µg ml^−1^, Acro Biosystems, MSN-H526x) and soluble anti-human CD28 (2 µg ml^−1^, 302943, BioLegend) in complete TexMACS GMP Medium for 24 h at 37 °C and 5% CO_2_. TRuC cells were then labeled with membrane stain (1:500 dilution, IsoPlexis) and either anti-human CD8-APC antibody (BioLegend, 301049) or anti-human CD4-APC antibody (BioLegend, 300537). Next, 30,000 live cells were loaded into a 32-plex human adaptive IsoCode Chip (IsoPlexis) Each condition was loaded in triplicate (*n* = 3 TRuC CD4^+^ cells and *n* = 3 TRuC CD8^+^ cells). Polyfunctionality of T cells defined as a cell co-secreting 2+ cytokines were analyzed by the IsoSpeak software across the seven functional groups: Th1 pro-inflammatory (GM-CSF, IFN-γ, IL-2, IL-12, TNF-α and TNF-β); Th2 pro-inflammatory (IL-4, IL-5, IL-7, IL-9 and IL-13); Chemoattractive (CCL11, IL-8, IP-10, MCP-1, MCP-4, MIP1-α, MIP-1β and RANTES); Regulatory (IL-10, IL-15, IL-22 and TGF-β1); Th17 pro-inflammatory (IL-1β, IL-6, IL-17A, IL-17F and IL-21); Cytolytic (granzyme B and perforin); and Other (sCD40L and sCD137). The PSI of T cells was computed using a pre-specified formula, defined as the percentage of polyfunctional cells multiplied by the sum of the mean fluorescence intensity (MFI) of the proteins secreted by those cells: PSI = (% polyfunctional cells in sample) × ∑(MFI of all 32 secreted proteins of the polyfunctional cells).

### ctDNA measurement

A post hoc analysis of circulating tumor DNA (ctDNA) was performed. Baseline and post-infusion venous blood samples were taken from patients using sodium heparin blood tubes and processed for plasma. Plasma samples (1–2.5 ml) were stored at –80 °C until assays were performed. All ctDNA extraction, processing and sequencing from plasma were performed in a CLIA-certified, CAP-accredited laboratory (Guardant Health) as previously described^39^ and analyzed using the 500-gene GuardantOMNI panel as previously described^40^.

### T cell clonotypic analysis

T cell clonotypic analysis was performed by Adaptive Biotechnologies using their ImmunoSEQ hsTCRB sequencing kit on TRuC-T cell product or baseline and post-infusion PBMC samples. In brief, gDNA was isolated and amplified using multiplexed primers and sequenced on Illumina NextSeq. Data analysis of TCR clonotype and abundance was performed using ImmunoSEQ Analyzer (Adaptive Biotechnologies). Compare patient’s infusion product to their underlying repertoire to detect changes in clonality, expansion and persistence in post-infusion samples, peripherally.

### Multiplex immunofluorescence assay

MultiOmyxTM technology was used to evaluate the expression of a panel of 14 biomarkers, including TIGIT, CD155, LAG3, CD4, CD56, CD3, CTLA4, CD8, PD-L1, PD-1, FoxP3, CD68 and TIM-3 and tumor segmentation marker PanCK on ovarian, cholangiocarcinoma and mesothelioma cancer FFPE samples. FFPE slides were evaluated by pathology (at NeoGenomics) for tissue annotation and selection. The selected tumor-relevant areas by pathology were used for staining and analysis. The staining was performed using a single 5-µM FFPE slide. Within each staining round, two cyanine dye-labeled (Cy3 and Cy5) antibodies were paired together and recognized two markers. The staining signal was then imaged and followed by novel dye inactivation, enabling repeated rounds of staining. For cell classification for all individual markers, proprietary deep-learning-based workflows were applied to identify individual cells and perform individual cell classification results. These results were combined to generate co-expression summaries and compute spatial distribution statistics for phenotypes of interest. For area quantification of PD-L1 or CD155, ImageJ software (NIH) was used to measure area of fluorescent marker expression within three high-powered fields divided by the total area. For visualization of each marker, individually established fluorescent minimum and maximum values were set and applied equally across all samples using the fluorescent look-up table (LUT).

### ADA detection by a bridging MSD assay

In the screening assay, human plasma samples are incubated for 1 h with a sulfo-tagged MH1 reagent (MH1 is the anti-mesothelin sdAb used in the gavo-cel TRuC) and then added onto a streptavidin plate coated with biotinylated MH1. After 1 h of incubation, the plate is washed to remove unbound material and read on the MSD Imager. The imager reads the ECL signal generated by the sulfo-tagged MH1 reagent and converts it to a relative light unit (RLU). The RLU level is directly proportional to the amount of anti-MH1 antibody in the plasma sample. If samples are found to be negative in the screening assay, testing is complete. Samples found to be positive in the screening assay are tested in a confirmatory assay in the presence/absence of MH1 to confirm specificity. If samples are negative in the confirmatory assay, testing is complete. Samples found to be positive in the confirmatory assay are tested in a titration assay to determine the titer at which positive detection is lost.

### Statistical analysis

The sample size throughout dose escalation was based on safety considerations and following a modified 3 + 3 dose-escalation/de-escalation schema. Descriptive statistics include calculations of mean, median, standard deviation, minimum and maximum values for continuous variables and frequency distributions for categorical variables. All AEs were categorized according to the ICH MedDR codes version 23.1 and graded according to CTCAE version 5.0, except for CRS, which was graded according to the ASTCT Consensus Grading Scale. AEs were analyzed using descriptive statistics and classified by organ system and preferred term. The Kaplan–Meier method was used for PFS and OS calculations. Non-parametric tests were used throughout. Kruskal–Wallis ANOVA was used for TCR-β sequencing data and cytokine analyses. Unpaired Mann–Whitney *t*-test was used for SMRP and MPF data. All phase 1 patients (*n* = 32) were analyzed for each assay where available. GraphPad Prism version 9.5 software was used to plot data.

### Reporting Summary

Further information on research design is available in the Nature Portfolio Reporting Summary linked to this article.

## Online content

Any methods, additional references, Nature Portfolio reporting summaries, source data, extended data, supplementary information, acknowledgements, peer review information; details of author contributions and competing interests; and statements of data and code availability are available at 10.1038/s41591-023-02452-y.

### Supplementary information

Supplementary Information Supplementary Tables 1 and 2 and Supplementary Figs. 1–7.

Reporting Summary

Supplementary Data Source data for Supplementary Figs. 3–6.

### Source data

Source Data Fig. Source data for Figs. 2c,d, 3c,d,f,g and 4a–d.

Source Data Extended Data Figures Source data for Extended Data Figs. 4a–c, 5d, 6b,c, 7a–g, 8a,b and 9a–g.

## Figures and Tables

**Extended Data Table 1:**
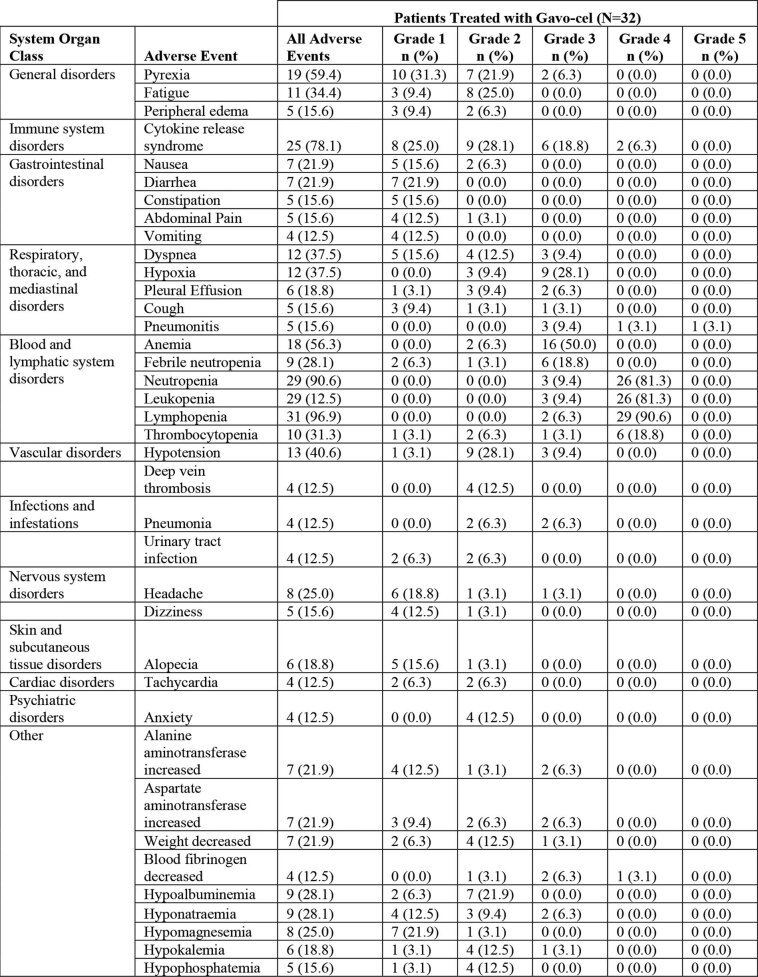
Treatment-emergent AEs reported in at least 10% of patients. All emergent AEs described in the table were deemed at least possibly related to protocol-defined therapy

## Data Availability

All data used in the analysis of the findings in the present study are included in the manuscript and the Supplementary Information. Pseudonymized participant data, including outcomes and relevant reported patient characteristics, will be shared as Supplementary Information. All requests for raw data and analyzed data should be directed to one of the corresponding authors: R.H., A.Q.-C. or D.H. Request for raw data and analyzed data will be reviewed by the corresponding authors to determine if the request is subject to any intellectual property or confidentiality considerations. Patient identities will not be revealed owing to patient confidentiality. Any data or material that can be shared will be done via a material transfer agreement. The TCR-β sequencing data are available in ImmuneACCESS (10.21417/RH2023NM). Source data are provided with this paper.
