# Supplementary material for: Mesothelin-targeting T cell receptor fusion construct cell therapy in refractory solid tumors: phase 1/2 trial interim results
Source: Nat Med. 2023 Jul 27;29(8):2099–109. doi: 10.1038/s41591-023-02452-y (PMC10427427; doi:10.1038/s41591-023-02452-y)
Supplement: Supplementary file 1 — Supplementary Tables 1 and 2 and Supplementary Figs. 1–7. [file 41591_2023_2452_MOESM1_ESM.pdf]

nature medicine

Article https://doi.org/10.1038/s41591-023-02452-y

Mesothelin-targeting T cell receptor fusion construct cell therapy in refractory solid tumors: phase 1/2 trial interim results

In the format provided by the authors and unedited

TABLE OF CONTENTS

SUPPLEMENTARY TABLES

Table S1. Detection of TRuC-T cells in serosal effusions by qPCR

Table S2. Anti-drug antibodies pre- and post- infusion of gavo-cel

SUPPLEMENTARY FIGURES

Supplementary Fig. 1, Mesothelin expression in tumor biopsies.

Supplementary Fig. 2, Graphical representation of change in metabolic tumor volume and total lesion glycolysis in a patient with MPM experiencing partial response.

Supplementary Fig. 3, Kinetics of plasma cytokine release post gavo-cel infusion.

Supplementary Fig. 4, Kinetics of plasma cytokine release post gavo-cel infusion.

Supplementary Fig. 5, Gavo-cel expansion in responders vs non-responders.

Supplementary Fig. 6, Quantitative imaging analysis of multiplex immunofluorescence assay.

Supplementary Fig. 7, Representative gating strategy used for analysis of flow cytometry data.

NR_REPORTING_SUMMARY

SOURCE DATA: SUPPLEMENTARY FIGURES

SUPPLEMENTARY TABLES

Table S1. Detection of TRuC-T cells in serosal effusions by qPCR

Copies/μg gDNA in Serosal effusion			Copies/μg gDNA in Blood		
Pt#	Days	Copies of DNA	Copies at proximal timepoint	Peak	Last day of detectable OPRE
7	10	44791.6	186.3 (day 10)	712.9 (day21)	day 66
14	16	19670.6	242.2 (day 21)	14359.7 (day 7)	day 29
20	76	343.7	289.1 (day 43)	5135.2 (day 9)	day 43
15	64	183.9	743.2 (day 57)	59425.3 (day 9)	day 394
25	58	148.9	97.0 (day 56)	100781.9 (day 7)	day 56
25	70	67.7	Not detected at day 86	100781.9 (day 7)	day 56

Table S2. Anti-drug antibodies pre- and post- infusion of gavo-cel¶.

Patient number	Visit Name	Day	ValueS	ValueC	ValueT
1	Baseline	-5	Negative	Negative	NA
	Week 8	49	Positive	Positive	8
3	Baseline	-12	Negative	Negative	NA
	Week 8	60	Negative	Negative	NA
6	Baseline	-12	Negative	Negative	NA
	Week 8	56	Negative	Negative	NA
7	Baseline	-56	Negative	Negative	NA
	Week 8	56	Negative	Negative	NA
8	Baseline	-8	Negative	Negative	NA
	Week 8	56	Negative	Negative	NA
11	Infusion	0	Negative	Negative	NA
	Week 8	56	Negative	Negative	NA
12	Infusion	0	Negative	Negative	NA
	Week 8	56	Negative	Negative	NA
13	Infusion	0	Negative	Negative	NA
	Week 8	58	Negative	Negative	NA
14	Infusion	0	Negative	Negative	NA
	Week 8	56	Negative	Negative	NA
15	Infusion	0	Negative	Negative	NA
	Week 8	57	Negative	Negative	NA
18	Infusion	0	Negative	Negative	NA
	Week 8	55	Negative	Negative	NA

19	Infusion	0	Negative	Negative	NA
	Week 8	56	Negative	Negative	NA
22	Infusion	0	Negative	Negative	NA
	Week 8	56	Negative	Negative	NA
24	Infusion	0	Negative	Negative	NA
	Week 8	56	Negative	Negative	NA
25	Infusion	0	Negative	Negative	NA
	Week 8	56	Negative	Negative	NA
28	Infusion	0	Negative	Negative	NA
	Week 8	63	Negative	Negative	NA
29	Infusion	0	Negative	Negative	NA
	Week 12	76	Negative	Negative	NA
30	Infusion	0	Negative	Negative	NA
	Week 8	56	Negative	Negative	NA
31	Infusion	0	Negative	Negative	NA
	Week 8	63	Negative	Negative	NA
32	Baseline	-15	Negative	Negative	NA
	Week 8	63	Negative	Negative	NA

¶Antibodies against the anti-MSLN MH1 binder moiety contained in the TRuC were measured in plasma pre- and post-infusion using a validated sandwich ELISA method. ValueS: screen result, ValueC: confirmatory result, ValueT: titration result.

SUPPLEMENTARY FIGURES

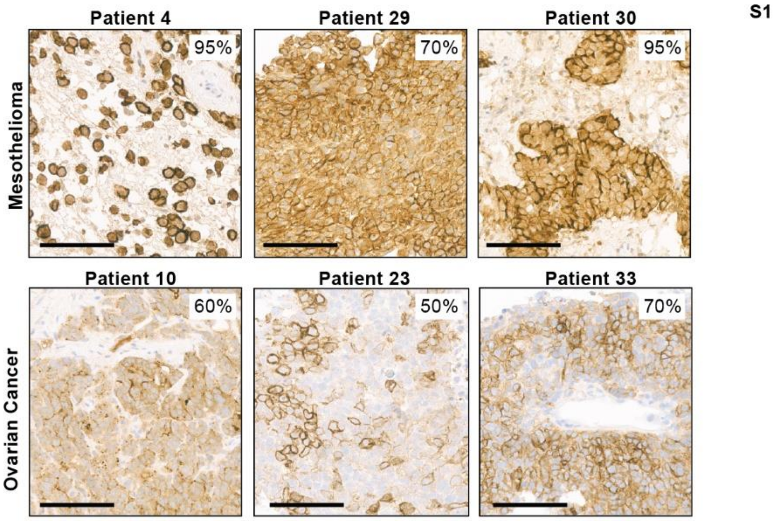

Supplementary Fig. 1, Mesothelin expression in tumor biopsies. Representative micrographs of mesothelin immunohistochemistry in **a,** mesothelioma and **b,** ovarian cancer patients. All patients enrolled to the study had mesothelin staining in the tumor compartment that surpassed the threshold of 50% membrane positivity of combined 2+ and 3+, scored by a trained pathologist. Percentage in each patient micrograph represent the proportion of viable tumor cells expressing membranous mesothelin combination 2+ and 3+ intensity. Experiment performed once on independent patient samples. Inset scale bar represents 100 µM.

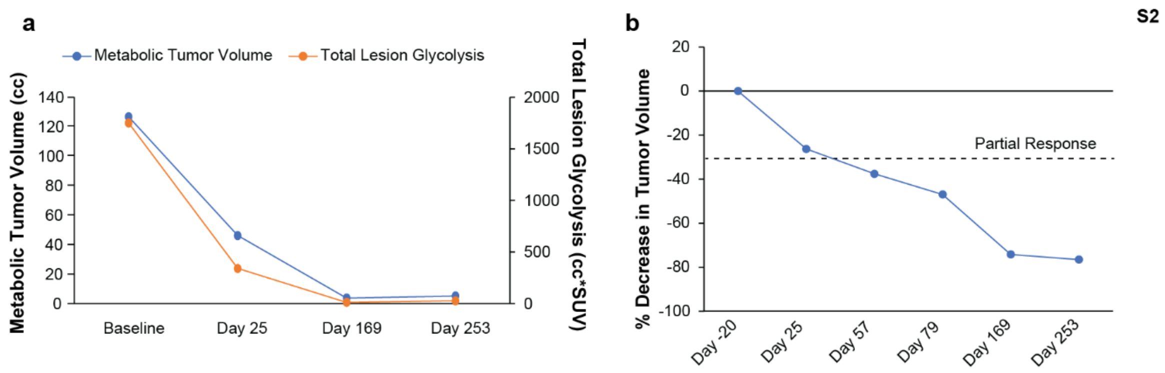

Supplementary Fig. 2, Graphical representation of change in metabolic tumor volume and total lesion glycolysis in a patient with MPM experiencing partial response. a, The metabolic tumor volume decreased from 126.7cc at baseline to 5.2cc at day 253. Total lesion glycolysis also went down from 1750.6cc*SUV to 27.7cc*SUV. **b,** Progressive decrease in the sum of target tumor lesions as measured by CT scans. The patient achieved a partial response by day 57 and on day 253 had a 76.5% decrease in the sum of tumor target lesions.

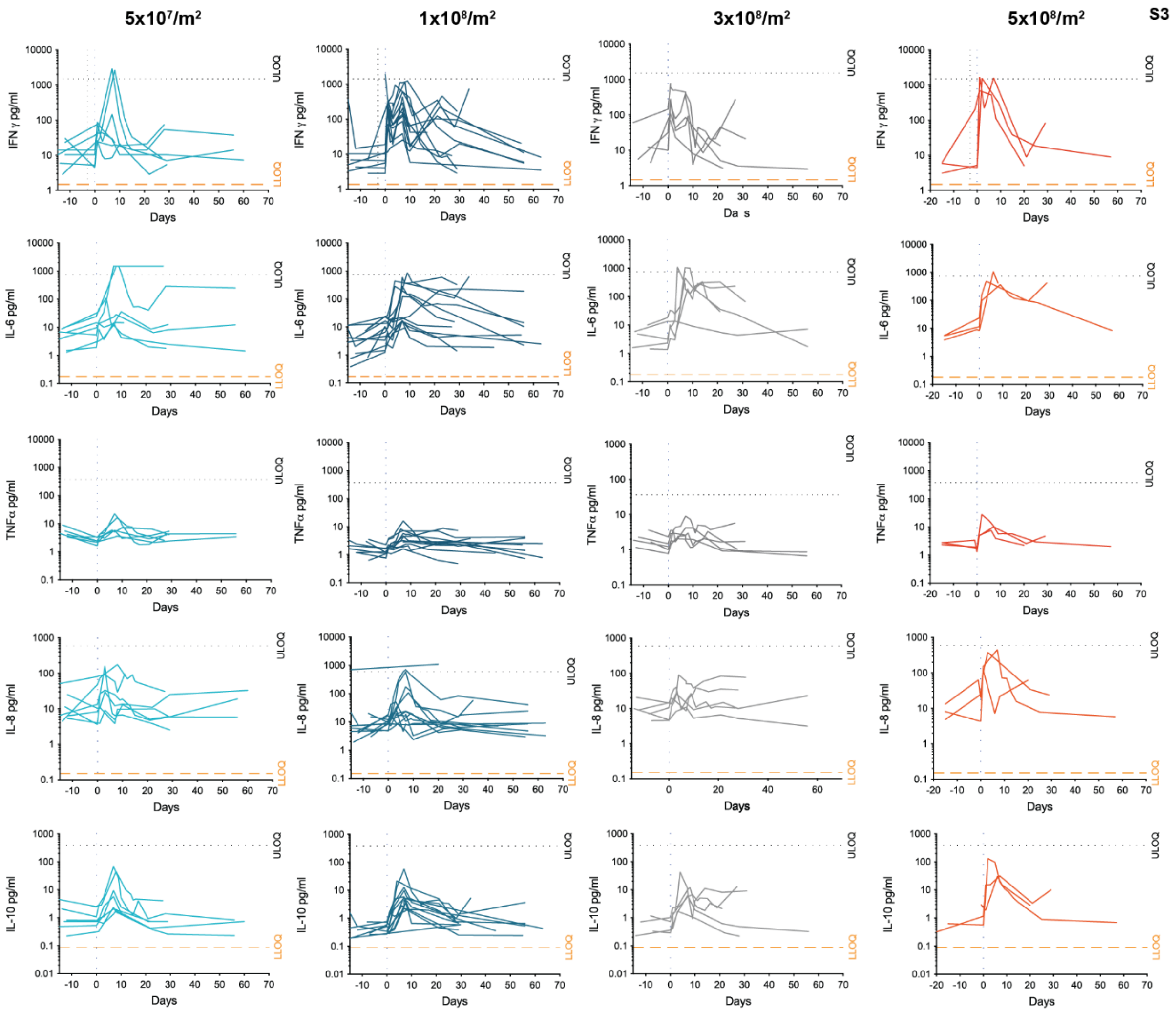

Supplementary Fig. 3, Kinetics of plasma cytokine release post gavo-cel infusion. Plasma levels of IFN-γ, IL-6, TNF-α, IL-8 and IL-10 by dose level for individual patients as measured longitudinally in the peripheral blood using a validated multiplexed immunoassay (Meso Scale Discovery).

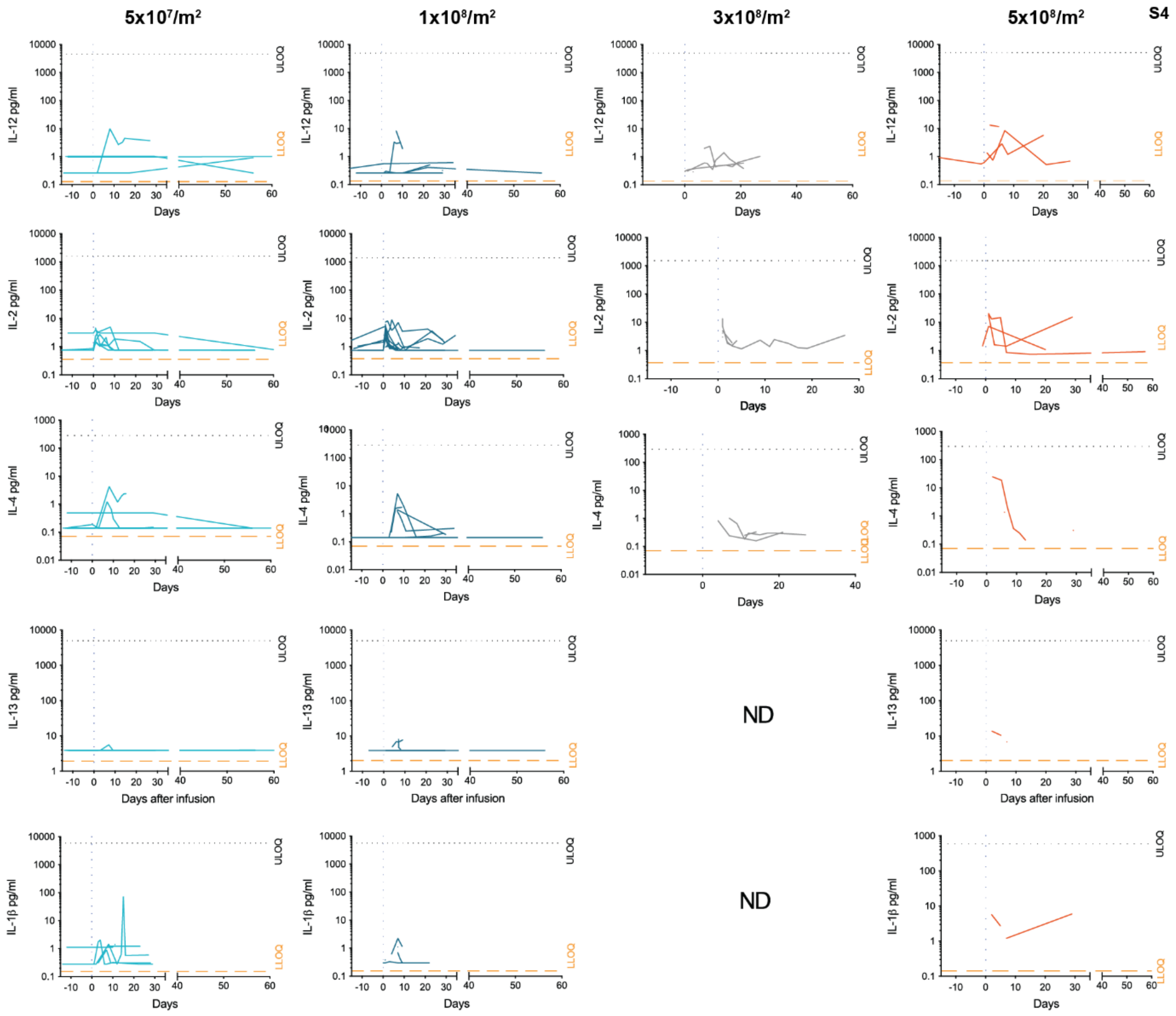

Supplementary Fig. 4, Kinetics of plasma cytokine release post gavo-cel infusion. Plasma levels of IL-12, IL-2, IL-4, IL-13 and IL-1β by dose level for individual patients as measured longitudinally in the peripheral blood using a validated multiplexed immunoassay (Meso Scale Discovery). N.D., Not detected.

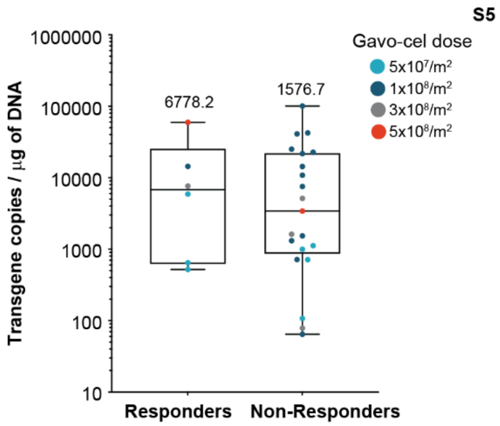

Supplementary Fig. 5, Gavo-cel expansion in responders vs non-responders. Comparison of transgene expression (C_{max}) by qRT PCR in responders by RECIST vs. non-responders. The correlation between peak vector transgene copies per microgram of gDNA after gavo-cel infusion and response assessment by RECIST version 1.1 criteria. Horizontal lines and boxes show the medians and interquartile ranges, respectively. (n=29 independent patient samples) For both box-plots; center line, box limits and whiskers represent the median, interquartile range and minima and maxima, respectively.

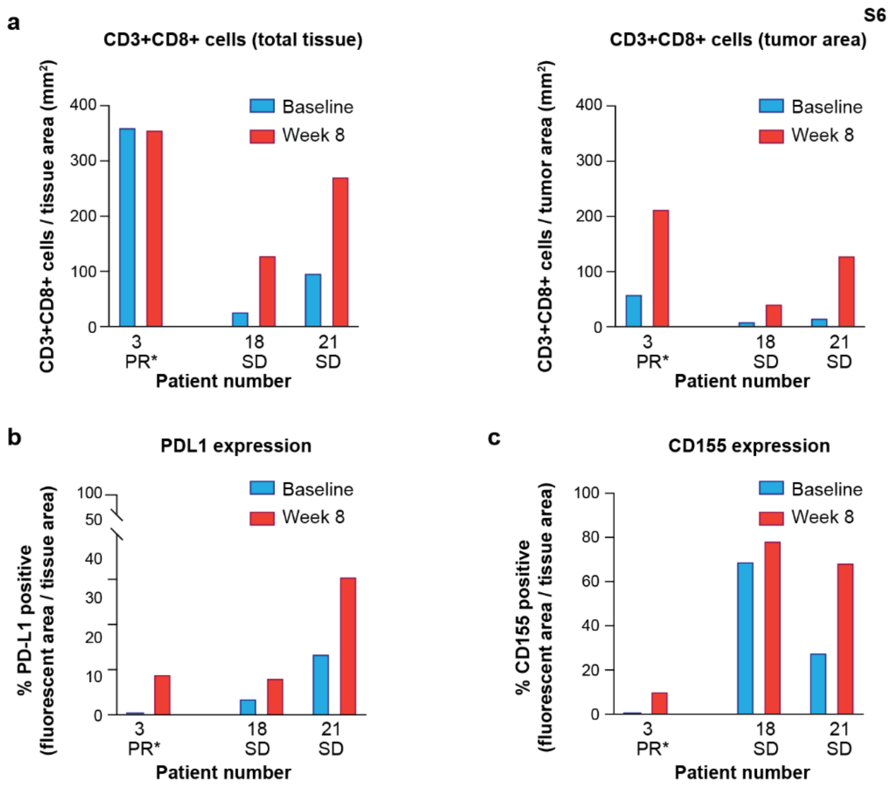

Supplementary Fig. 6, Quantitative imaging analysis of multiplex immunofluorescence assay. Multiplex immunofluorescent staining was performed for cytokeratin (tumor marker), CD3 (pan-T cell marker), PD-L1 and CD155 in malignant pleural mesothelioma (MPM) tumor biopsies taken at baseline and at week 8 post gavo-cel infusion. **a,** Numbers of CD3+CD8+ T cells in total tissue vs. tumor area tissue. **b,** Percent positive staining by surface area for PD-L1. **c,** Percent positive staining by surface area for CD155. PR, partial response; SD, stable disease

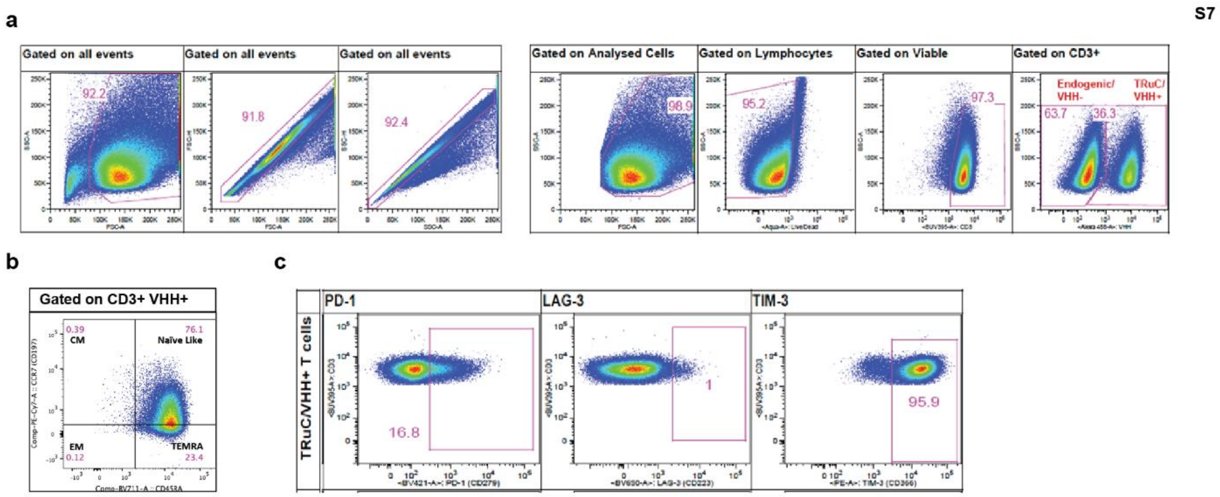

Supplementary Fig. 7, Representative gating strategy used for analysis of flow cytometry data. a, Overall gating strategy used for identifying TRuC T cells. **b,** after using the strategy defined in (a), CD3+ VHH+ T cells were further defined into memory phenotypes (CM= central memory, EM= effector memory, TEMRA= effector memory cells re-expressing CD45RA). **c,** after using the strategy defined in (a), CD3+ VHH+ T cells were analyzed for expression of PD-1, LAG-3 and TIM-3.
