# Supplementary material for: Mesothelin-targeting T cell receptor fusion construct cell therapy in refractory solid tumors: phase 1/2 trial interim results
Source: Nat Med. 2023 Jul 27;29(8):2099–109. doi: 10.1038/s41591-023-02452-y (PMC10427427; doi:10.1038/s41591-023-02452-y)
Supplement: Supplementary file 2 — Reporting Summary [file 41591_2023_2452_MOESM2_ESM.pdf]

nature portfolio

Corresponding author(s): Raffit Hassan, M.D., Alfonso Quintás-Cardama, M.D., and David Hong, M.D.

Last updated by author(s): 2023/5/31

Reporting Summary

Nature Portfolio wishes to improve the reproducibility of the work that we publish. This form provides structure for consistency and transparency in reporting. For further information on Nature Portfolio policies, see our Editorial Policies and the Editorial Policy Checklist.

Statistics

For all statistical analyses, confirm that the following items are present in the figure legend, table legend, main text, or Methods section.

n/a	Confirmed	
☐	☒	The exact sample size (*n*) for each experimental group/condition, given as a discrete number and unit of measurement
☐	☒	A statement on whether measurements were taken from distinct samples or whether the same sample was measured repeatedly
☐	☒	The statistical test(s) used AND whether they are one- or two-sided<br>*Only common tests should be described solely by name; describe more complex techniques in the Methods section.*
☒	☐	A description of all covariates tested
☒	☐	A description of any assumptions or corrections, such as tests of normality and adjustment for multiple comparisons
☐	☒	A full description of the statistical parameters including central tendency (e.g. means) or other basic estimates (e.g. regression coefficient) AND variation (e.g. standard deviation) or associated estimates of uncertainty (e.g. confidence intervals)
☐	☒	For null hypothesis testing, the test statistic (e.g. *F*, *t*, *r*) with confidence intervals, effect sizes, degrees of freedom and *P* value noted<br>*Give P values as exact values whenever suitable.*
☒	☐	For Bayesian analysis, information on the choice of priors and Markov chain Monte Carlo settings
☒	☐	For hierarchical and complex designs, identification of the appropriate level for tests and full reporting of outcomes
☒	☐	Estimates of effect sizes (e.g. Cohen's *d*, Pearson's *r*), indicating how they were calculated

Our web collection on statistics for biologists contains articles on many of the points above.

Software and code

Policy information about availability of computer code

Data collection	LSR flow cytometer (BD Biosciences), Applied Biosystems ViiA7 Real Time PCR System (Life Technologies),Illumina NextSeq, MultiOmyxTM technology, MesoScale Discovery (MSD) Imager, IsoLight (IsoPlexis)
Data analysis	FlowJo software (BD Biosciences) 10.8.1, IsoSpeak software 2.7.0.0, ImmunoSEQ Analyzer (Adaptive Biotechnologies) 2.0, ImageJ software (NIH, Bethesda, Maryland) 1.54 p4, GraphPad Prism 9.5, BD FACSDiva V9, GuavaSoft 3.3

For manuscripts utilizing custom algorithms or software that are central to the research but not yet described in published literature, software must be made available to editors and reviewers. We strongly encourage code deposition in a community repository (e.g. GitHub). See the Nature Portfolio guidelines for submitting code & software for further information.

Data

Policy information about availability of data

All manuscripts must include a data availability statement. This statement should provide the following information, where applicable:

- Accession codes, unique identifiers, or web links for publicly available datasets
- A description of any restrictions on data availability
- For clinical datasets or third party data, please ensure that the statement adheres to our policy

All data used in the analysis of the findings in the present study are included in the manuscript and the Supplementary Information. Pseudonymized participant data, including outcomes and relevant reported patient characteristics, will be shared as Supplementary Information. All requests for raw data and analyzed data

should be directed to either of the corresponding authors: Dr. Raffit Hassan, M.D., Dr. Alfonso Quintás-Cardama, M.D., David Hong, M.D. Request for raw data and analyzed data will be reviewed by the corresponding authors to determine if the request is subject to any intellectual property or confidentiality considerations. The patient identity will not be revealed due to patient confidentiality. Any data or material that can be shared will be done via Material Transfer Agreement. The TCRbeta sequencing data is available in ImmuneACCESS. DOI: 10.21417/RH2023NM; URL: https://clients.adaptivebiotech.com/pub/hassan-2023-nm

Human research participants

Policy information about studies involving human research participants and Sex and Gender in Research.

Reporting on sex and gender

Participants from both the sexes were included in this study. No sex- based analysis was performed.
Neither sex nor gender was considered in the study design. Patients were enrolled on a "first come first served" basis. Sex/gender was determined based on self-report.

Population characteristics

Inclusion criteria:
Eligibility for the study required that patients met all of the following criteria:
1. Voluntarily agreed to participate by giving written informed consent in accordance with International Conference on Harmonization (ICH) Good Clinical Practice (GCP) guidelines and applicable local regulations.
2. Agreement to abide by all protocol required procedures, including study related assessments, and management by the treating institution for the duration of the study and long-term follow-up.
3. Age ≥ 18 years of age at the time the informed consent is signed.
4. Pathologically confirmed diagnosis of either MPM, serous ovarian adenocarcinoma (patients with serous fallopian tube or primary peritoneal cancers were also eligible), cholangiocarcinoma, or NSCLC.
5. Tumor was pathologically reviewed at a sponsor designated central laboratory with confirmed positive mesothelin (MSLN) expression on ≥ 50% of tumor cells with 2+ and/or 3+ staining intensity by immunohistochemistry. A fresh biopsy for confirmation of mesothelin expression was required at baseline (if not done at pre-screening) prior to gavo-cel administration.
6. Patient had advanced (i.e., metastatic, or unresectable) cancer.
7. Patient had at least 1 lesion that met evaluable and measurable criteria defined by Response Evaluation Criteria in Solid Tumors (RECIST) version 1.1.
8. Prior to gavo-cel infusion, patients must have received at least 1 systemic standard of care therapy for metastatic or unresectable disease and have failed U.S. FDA approved agents for their disease (e.g., PARP inhibitors for BRCA1/2 mutated ovarian cancer or osimertinib for patients with EGFR T790M mutation). Patients with newly diagnosed cholangiocarcinoma could receive gavo-cel infusion if they elected not to pursue frontline standard of care therapy.
9. Eastern Cooperative Oncology Group (ECOG) performance status 0 or 1.
10. Negative rapid influenza diagnostic test and/or a respiratory viral panel (as per Institutional guidelines) within 14 days prior to the planned gavo-cel infusion. Respiratory viral panel should be performed according to institutional guidelines and include coronavirus disease 2019 (Covid-19; SARS-CoV-2). If patient was symptomatic or tested positive, the gavo-cel infusion was delayed until the patient was asymptomatic and deemed fit for infusion by the treating physician.
11. Left ventricular ejection fraction ≥ 45% as measured by resting echocardiogram, with no clinically significant pericardial effusion.
12. Female patients of childbearing potential (FCBP) must have a negative urine or serum pregnancy test (FPCP is defined as premenopausal and not surgically sterilized). FPCP must agree to use effective birth control or to abstain from heterosexual activity throughout the study, starting on the day of first dose of lymphodepleting chemotherapy through 12 months post gavo-cel infusion or for 4 months after there is no evidence of persistence of gene modified cells in the blood, whichever is longer. Effective contraceptive methods include intra-uterine device, oral or injectable hormonal contraception, or 2 adequate barrier methods (e.g., diaphragm with spermicide, cervical cap with spermicide, or female condom with spermicide). Spermicides alone are not an adequate method of contraception. Male patients must be surgically sterile or agree to use a double barrier contraception method or abstain from heterosexual activity with a female of childbearing potential starting at the first dose of protocol-defined treatment and for 4 months thereafter or longer (if indicated in the country specific monograph/label for cyclophosphamide).
13. Patient must have adequate organ function according to the following laboratory values:
Hematological
Absolute neutrophil count ≥ 1 × 109/L (without growth factor support)
Absolute lymphocyte count ≥ 0.3 × 109/L
Platelets ≥ 100 × 109/L
Hemoglobin ≥ 90 g/L (without transfusion support within 7 days prior to protocol defined therapy)
Coagulation
Prothrombin time ≤ 1.5 × upper limit of normal (ULN)
Partial thromboplastin time ≤ 1.5 × ULN
Renal
Creatinine clearance (Cockcroft–Gault formula) ≥ 40 mL/min
Hepatic
Serum total bilirubin ≤ 2 × ULN (unless patient has documented Gilbert's syndrome or unless secondary to bile duct obstruction by tumor)
Alanine aminotransferase ≤ 2.5 × ULN or ≤ 5 × ULN if documented liver metastasis
Aspartate aminotransferase ≤ 2.5 × ULN or ≤ 5 × ULN if documented liver metastasis

Exclusion Criteria:
Patients meeting any of the following criteria were not eligible for participation in the study:
1. Inability to follow the study procedures.
2. Known or suspected noncompliance, drug, or alcohol abuse.
3. Participation in another study with investigational drug within the 28 days or 5 half-lives of the drug, whichever is shorter, preceding and during the present study.

4. Patient is pregnant (or intends to become pregnant during the course of the study) or breastfeeding.
5. Patient has received the following treatment prior to initiating protocol-defined therapy with either lymphodepletion or gavo-cel:
i. Cytotoxic chemotherapy within 3 weeks of gavo-cel infusion.
ii. Corticosteroids: therapeutic doses of steroids must be stopped at least 2 weeks prior to gavo-cel infusion. Inhaled corticosteroids are not exclusionary.
iii. Immunosuppression: any other immunosuppressive drugs (e.g., methotrexate, mycophenolate) must be stopped ≥ 4 weeks prior to first protocol defined treatment.
iv. Use of an anti-cancer vaccine within 2 months in the absence of tumor response or 6 months if responding.
v. Any previous gene therapy using an integrating vector.
vi. Tyrosine kinase inhibitors within 72 hours.
vii. Any previous allogeneic hematopoietic stem cell transplant.
viii. Investigational treatment or clinical trial within 4 weeks or 5 half-lives of investigational product, whichever is shorter.
ix. Radiotherapy to the target lesions within 3 months prior to lymphodepleting chemotherapy unless palliative radiotherapy to non-targeted lesions.
x. Current anticoagulative therapy (excluding deep vein thrombosis prophylaxis).
xi. Immunotherapy (monoclonal antibody therapy, checkpoint inhibitors) within 4 weeks.
6. Toxicity from previous anti-cancer therapy that had not recovered to ≤ grade 1 (except for non-clinically significant toxicities, e.g., alopecia, vitiligo). Grade 2 toxicities that are deemed stable or irreversible (e.g., peripheral neuropathy) are non-exclusionary.
7. History of allergic reactions attributed to compounds of similar chemical or biologic composition to fludarabine, cyclophosphamide, or other agents used in the study.
8. History of autoimmune or immune-mediated disease such as multiple sclerosis, lupus, rheumatoid arthritis, inflammatory bowel disease, or small vessel vasculitis.
9. Major surgery (other than diagnostic surgery) within 4 weeks prior to first protocol defined therapy, minor surgery including diagnostic surgery within 2 weeks (14 days) excluding central iv port placements and needle aspirate/core biopsies. Radiofrequency ablation or transcatheter arterial chemoembolization within 6 weeks prior to enrollment.
10. Leptomeningeal disease, carcinomatous meningitis, or symptomatic central nervous system (CNS) metastases: patients are eligible if they have recovered from the acute effects of radiation therapy or surgery prior to study entry, and a) have no evidence of brain metastases post treatment or b) are asymptomatic, have discontinued corticosteroid or anti-seizure therapy for metastases for at least 4 weeks and have radiographically stable CNS metastases (no growth, edema, or shift for at least 3 months prior to study entry).
11. Any other prior or concurrent malignancy with the exception of treated basal cell or squamous cell carcinoma, in situ carcinoma of the cervix or breast, stage 0 or 1 melanoma completely resected more than 12 months prior to enrollment, successfully treated organ-confined prostate cancer, other malignancies completely resected and in remission for more than 5 years.
12. Electrocardiogram (ECG) showing a clinically significant abnormality at screening.
13. Uncontrolled intercurrent illness including active infection, clinically significant cardiac disease (e.g., congestive heart failure New York Heart Association class 3 or class 4, significant arrhythmia, acute coronary syndrome), interstitial lung disease, liver cirrhosis, or primary sclerosing cholangitis.
14. Active infection with human immunodeficiency virus (HIV), hepatitis B virus, hepatitis C virus (HCV), or human T-lymphotropic virus (HTLV).

Recruitment

Based on eligibility criteria, patients were recruited on first come first serve basis. Informed consent was obtained from all participants in the clinical trial, as stated in the clinical trial protocol. Participants were not compensated for their participation in the clinical trial. We do not identify any bias that could have impacted the results. Patients were recruited between June, 2019, till February 28th, 2022.

Ethics oversight

The trial was approved by the Institutional Review Board of the participating centers and all the patients provided written informed consent.

The seven participating centers are:

1. Thoracic and GI Malignancies Branch, Center for Cancer Research, National Cancer Institute, National Institutes of Health, Bethesda, MD, USA

2. Princess Margaret Cancer Centre, Toronto, Canada

3. Department of Medical Oncology, Memorial Sloan Kettering Cancer Center, Weill Cornell Medical College, New York, NY, USA

4. Division of Hematology/Oncology, Department of Medicine, University of California, San Francisco, CA, USA

5. Sarah Cannon Cancer Center, Nashville, TN, USA

6. Hospital of the University of Pennsylvania, Abramson Cancer Center, Philadelphia, PA, USA

7. Department of Investigational Cancer Therapeutics, University of Texas M.D. Anderson Cancer Center, Houston, TX, USA

Note that full information on the approval of the study protocol must also be provided in the manuscript.

Field-specific reporting

Please select the one below that is the best fit for your research. If you are not sure, read the appropriate sections before making your selection.

☒ Life sciences ☐ Behavioural & social sciences ☐ Ecological, evolutionary & environmental sciences

For a reference copy of the document with all sections, see nature.com/documents/nr-reporting-summary-flat.pdf

Life sciences study design

All studies must disclose on these points even when the disclosure is negative.

Sample size	Sample size is based on patient enrollment. Thirty two-patients were enrolled in this study.
Data exclusions	All thirty-two patients who received gavo-cel infusion were evaluated for safety. For translational studies, patient samples with available data were included with sample size (n) mentioned in the figure legends
Replication	This is a clinical trial and individual patient serve as biological replicate.
Randomization	This is a single-arm study, therefore randomization is not required.
Blinding	This is an open -labeled trial, hence blinding is not required.

Reporting for specific materials, systems and methods

We require information from authors about some types of materials, experimental systems and methods used in many studies. Here, indicate whether each material, system or method listed is relevant to your study. If you are not sure if a list item applies to your research, read the appropriate section before selecting a response.

Materials & experimental systems

n/a	Involved in the study	
☐	☒	Antibodies
☒	☐	Eukaryotic cell lines
☒	☐	Palaeontology and archaeology
☒	☐	Animals and other organisms
☐	☒	Clinical data
☒	☐	Dual use research of concern

Methods

n/a	Involved in the study	
☒	☐	ChIP-seq
☐	☒	Flow cytometry
☒	☐	MRI-based neuroimaging

Antibodies

Antibodies used

CD3-BUV395 (BD Biosciences, cat#564001, clone SK7), 1:50 dilution
CD4-BUV496 (BD Biosciences, cat#612936, clone SK3), 1:25 dilution
CD8-APC-Cy7 (BD Biosciences, cat#348793, clone SK1), 1:100 dilution
CD366/TIM3-PE (BD Biosciences, cat#565570, clone 7D3), 1:400 dilution
CD27-BV605 (BioLegend,cat#302829, clone O323), 1:12.5 dilution
CD45RA-BV711 (BioLegend, cat#304137, clone HI100), 1:400 dilution
CD45RO-Alexa-700 (BioLegend, cat#304217, clone UCHL1), 1:25 dilution
CCR7-PE-Cy7 (BioLegend, cat#353225, clone G043H7), 1:25 dilution
CD95-BV785 (Biolegend, cat#305645, clone DX2), 1:100 dilution
CD279/PD-1-BV421 (BioLegend, cat#329919, clone EH12.2H7), 1:25 dilution
CD223/LAG-3 BV650 (BioLegend, cat#369315, clone 11C3C65), 1:50 dilution
and an anti-VHH-AF488 for TRuC detection (Genscript, cat#A01862, clone 96A3F5), 1:400 dilution

Validation

All commercially available antibodies were validated and routinely tested according to the manufacturer's instruction.
All the above listed antibodies were used on a immunophenotyping flow panel that was set-up and characterized by a third-party CRO. This included, antibody titration, FMO, assay precision tests, stability of antibodies and LLOQ determination. In addition all antibodies were tested on both healthy donor PBMCs and TRuC T cells prior to use on patient PBMCs

Clinical data

Policy information about clinical studies

All manuscripts should comply with the ICMJE guidelines for publication of clinical research and a completed CONSORT checklist must be included with all submissions.

Clinical trial registration	Clinical Trials.gov NCT03907852
Study protocol	The clinical protocol is included.
Data collection	Data was collected from eligible patients recruited between May 2019 and May 2022 in 7 participating centers in the US. The cut-off date for data analysis was September 9th, 2022. Peripheral blood and biopsies were collected based on Study calendar.
Outcomes	The primary objective was to evaluate safety and to determine the RP2D of gavo-cel. Secondary objectives included efficacy, by determining overall response rate (ORR), disease control rate (DCR), time to response (TTR), duration of response (DoR) and T cell kinetics.

Flow Cytometry

Plots

Confirm that:

☒ The axis labels state the marker and fluorochrome used (e.g. CD4-FITC).

☒ The axis scales are clearly visible. Include numbers along axes only for bottom left plot of group (a 'group' is an analysis of identical markers).

☒ All plots are contour plots with outliers or pseudocolor plots.

☒ A numerical value for number of cells or percentage (with statistics) is provided.

Methodology

Sample preparation	Human PBMCs were isolated from blood drawn into CPT tubes. Washed PBMCs were counted, aliquoted and cyropreserved in LN2 until use.
Instrument	BD LSR Fortessa was used to collect data.
Software	BD FACSDiva software version 9.0 was used to collect data and Flowjo software version 10.8.1 was used to analyze the data.
Cell population abundance	Viable (PBMC) cells were counted before acquisition by means of the automated guava counting process using the Viacount Reagent. (GuavaEasyCyte HT and GuavaSoft v3.3)
Gating strategy	FMO samples were used to define positive and negative populations in the initial experiments. FSC-A/SSC-A were used to eliminate debris and FSC-A/FSC-H were used to eliminate doublet populations, Live/Dead was used to identify viable cells.

☒ Tick this box to confirm that a figure exemplifying the gating strategy is provided in the Supplementary Information.
